# Protease-independent control of parthanatos by HtrA2/Omi

**DOI:** 10.1007/s00018-023-04904-7

**Published:** 2023-08-18

**Authors:** Jonas Weiß, Michelle Heib, Thiemo Korn, Justus Hoyer, Johaiber Fuchslocher Chico, Susann Voigt, Tomas Koudelka, Andreas Tholey, Dieter Adam

**Affiliations:** 1 Institut für Immunologie, Christian-Albrechts-Universität zu Kiel, Michaelisstr. 5, 24105 Kiel, Germany; 2 Institut für Experimentelle Medizin, Christian-Albrechts-Universität zu Kiel, Niemannsweg 11, 24105 Kiel, Germany

**Keywords:** HtrA2/Omi, Parthanatos, Proteolysis, Cell death

## Abstract

HtrA2/Omi is a mitochondrial serine protease with ascribed pro-apoptotic as well as pro-necroptotic functions. Here, we establish that HtrA2/Omi also controls parthanatos, a third modality of regulated cell death. Deletion of HtrA2/Omi protects cells from parthanatos while reconstitution with the protease restores the parthanatic death response. The effects of HtrA2/Omi on parthanatos are specific and cannot be recapitulated by manipulating other mitochondrial proteases such as PARL, LONP1 or PMPCA. HtrA2/Omi controls parthanatos in a manner mechanistically distinct from its action in apoptosis or necroptosis, i.e., not by cleaving cytosolic IAP proteins but rather exerting its effects without exiting mitochondria, and downstream of PARP-1, the first component of the parthanatic signaling cascade. Also, previously identified or candidate substrates of HtrA2/Omi such as PDXDC1, VPS4B or moesin are not cleaved and dispensable for parthanatos, whereas DBC-1 and stathmin are cleaved, and thus represent potential parthanatic downstream mediators of HtrA2/Omi. Moreover, mass-spectrometric screening for novel parthanatic substrates of HtrA2/Omi revealed that the induction of parthanatos does not cause a substantial proteolytic cleavage or major alterations in the abundance of mitochondrial proteins. Resolving these findings, reconstitution of HtrA2/Omi-deficient cells with a catalytically inactive HtrA2/Omi mutant restored their sensitivity against parthanatos to the same level as the protease-active HtrA2/Omi protein. Additionally, an inhibitor of HtrA2/Omi’s protease activity did not confer protection against parthanatic cell death. Our results demonstrate that HtrA2/Omi controls parthanatos in a protease-independent manner, likely via novel, unanticipated functions as a scaffolding protein and an interaction with so far unknown mitochondrial proteins.

**Supplementary Information:**

The online version contains supplementary material available at 10.1007/s00018-023-04904-7.

## Introduction

The mitochondrial serine protease HtrA2/Omi is a homolog of the bacterial HtrA/DegP protein from *Escherichia coli* and a member of the evolutionarily conserved High Temperature Requirement A (HtrA) protease chaperone family [1]. HtrA2/Omi is expressed from a nuclear gene as a 49-kDa proenzyme with an N-terminal mitochondrial localization signal that mediates translocation of HtrA2/Omi to the mitochondrial intermembrane space (IMS) [2, 3]. Although predominantly localized to the IMS [4], a fraction of the endogenous HtrA2/Omi pool has also been described in the endoplasmic reticulum and in the nucleus [5, 6]. In addition to the N-terminal mitochondrial targeting sequence, the full-length HtrA2/Omi protein consists of a transmembrane domain which anchors the protease in the inner mitochondrial membrane, a binding motif for inhibitor of apoptosis proteins (IAPs), a serine protease domain, and a PDZ domain at the C-terminus which acts as a protein–protein interaction module and regulates substrate binding and HtrA2/Omi activity [7, 8]. Within the mitochondria, the HtrA2/Omi proenzyme is proteolytically processed to its mature 36-kDa form, which is localized solubly in the IMS [9]. There, as a component of the mitochondrial quality control system, HtrA2/Omi degrades misfolded mitochondrial proteins, and thereby contributes to the maintenance of mitochondrial homeostasis [10]. In addition, HtrA2/Omi counteracts the degradation of mitochondria via mitophagy [11] and appears to be important for maintaining the cristae architecture of mitochondria [12]. Furthermore, as a positive regulator of autophagy, HtrA2/Omi facilitates the degradation of mutant proteins involved in neurodegenerative diseases [13]. Consistent with this protective function, deletion of HtrA2/Omi or mutations affecting its activity have been associated with neurodegeneration and Parkinson’s disease in mouse models [14] and in patients [15]. As a chaperone protein, HtrA2/Omi prevents the aggregation of amyloid β1-42, a major component of neurotoxic deposits in the brains of Alzheimer’s disease patients, in a protease-independent manner [16]. Conversely, however, an increased proteolytic activity of HtrA2/Omi is associated with Alzheimer’s dementia and with hypoxia-induced neuronal and cardiac muscle cell death [17–19]. Moreover, a role of HtrA2/Omi as a tumor suppressor in malignancies is discussed [11]. Recently, HtrA2/Omi has also been implicated as a diagnostic biomarker for hepatocellular carcinoma [20].

Apoptotic stimuli induce the release of HtrA2/Omi from mitochondria into the cytosol [2–4, 21]. There, it binds and cleaves the IAPs XIAP, cIAP1, cIAP2, and Apollon/BRUCE, neutralizing their inhibitory effect on caspases and enhancing apoptosis [1, 4, 22–24]. In addition, HtrA2/Omi promotes apoptosis by degradation of the anti-apoptotic proteins ped/pea-15 [25] and HAX-1 [26], by cleavage of the protein WT1 [27], and in the nucleus by proteolysis of the p73 protein, which stimulates transcription of the proapoptotic bax gene [28].

Early studies already suggested that HtrA2/Omi is also involved in non-apoptotic, caspase-independent forms of cell death [4, 29–32]. In our own work, using proteomic screens and functional approaches, we have previously identified HtrA2/Omi as a key component of both tumor necrosis factor (TNF)- and TNF-related apoptosis-inducing ligand (TRAIL)-induced necroptosis and demonstrated that pharmacological inhibition or genetic ablation of HtrA2/Omi protects against necroptosis [33, 34]. Currently, the exact molecular mechanism by which HtrA2/Omi is integrated into the necroptotic signaling cascade remains unknown. As one possibility, Zhang et al. recently proposed that HtrA2/Omi enhances necroptosis by degrading RIPK1 [35].

Parthanatos is a caspase-independent, non-apoptotic form of cell death that occurs in most neurological and neurodegenerative diseases such as amyotrophic lateral sclerosis [36], Parkinson’s disease [37, 38], stroke [39], Huntington’s disease [40], and Alzheimer’s disease [41] where it is responsible for neuronal loss [42]. It has long but erroneously been postulated that parthanatos is an integral component of necroptosis [43]. In own previous work, we have refuted this hypothesis and clearly demonstrated that parthanatos and necroptosis represent two distinct and independent pathways of regulated necrosis [44]. Parthanatos is triggered by oxidative stress, nitric oxide production, or massive DNA damage. This leads to hyperactivation of the DNA repair enzyme poly(ADPribose) (PAR) polymerase 1 (PARP-1), and subsequently to massive synthesis of PAR polymers from NAD^+^ and rapid depletion of intracellular NAD^+^ and ATP pools [42]. However, contrary to initial assumptions, it is not the loss of intracellular NAD^+^ and ATP that is critical for cell death, but the PAR polymer itself [45]. Similarly, the loss of NAD^+^ and ATP is not due to the massive synthesis of PAR [46], but rather to the binding of PAR to hexokinase and an inhibition of glycolysis [47, 48]. In the next step of the parthanatic signaling cascade, the mitochondrial protein apoptosis inducing factor mitochondria associated 1 (AIFM1) is released into the cytoplasm. In previous studies, AIFM1 release was reported to depend on its cleavage by calpain/cathepsin proteases [49–52], but this was later refuted [53, 54]. Nowadays, it is believed that the newly generated PAR polymers interact with a pool of AIFM1 that is located on the outer surface of the mitochondria [55], thereby causing its release into the cytosol [56]. In the cytosol, AIFM1 binds to macrophage migration inhibitory factor (MIF) and transports MIF into the nucleus, where AIFM1 and MIF form a complex with histone H2AX and cyclophilin A proteins. In this complex, MIF cleaves genomic DNA into large fragments via its nuclease activity, leading to the parthanatic death of the cell [57].

As detailed above, both HtrA2/Omi and parthanatos have important functions in the pathophysiology of neurodegenerative diseases. In addition, mitochondria are central components in parthanatic cell death, and HtrA2/Omi, as a mitochondrial protease, is essential for mitochondrial homeostasis. Nevertheless, to the best of our knowledge, a possible role of HtrA2/Omi in parthanatos has remained uninvestigated to date. Here, we establish its relevance as a critical mediator of this form of regulated cell death.

## Results

### Deletion of HtrA2/Omi protects from parthanatos

**Fig. 1:**
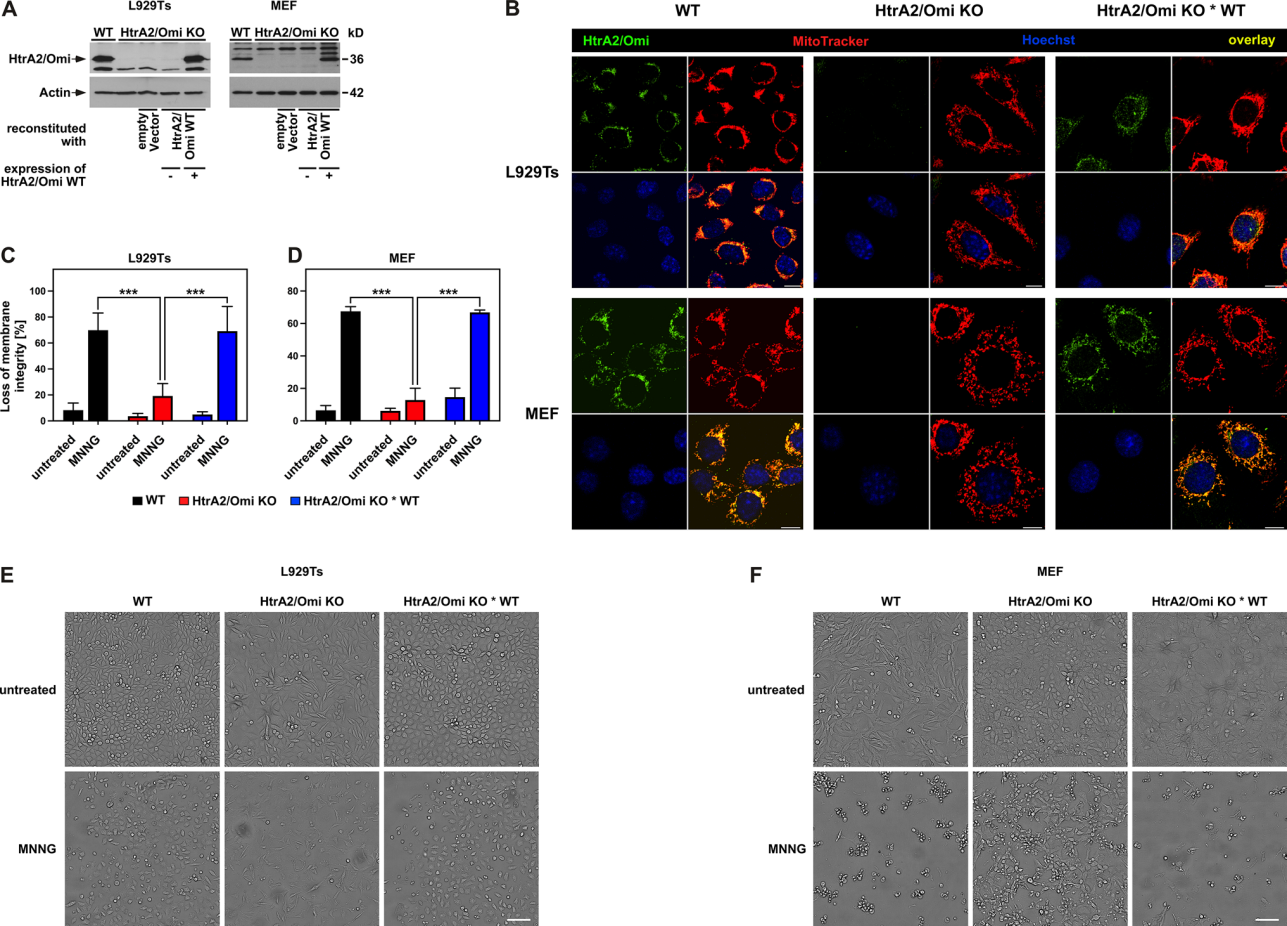
Role of HtrA2/Omi in parthanatos. **A** The presence or absence of HtrA2/Omi was verified by Western blot in parental L929Ts cells and MEF (WT), together with HtrA2/Omi-deficient cells (HtrA2/Omi KO) and HtrA2/Omi-deficient cells that had been reconstituted with WT HtrA2/Omi. Cells reconstituted with empty vector and cells where the reconstitution had not led to expression of HtrA2/Omi were originally included in this analysis and are shown for completeness. Detection of actin served as control for equal loading. **B** Additionally, the presence or absence of HtrA2/Omi was validated in L929Ts cells and in MEF [parental cells (WT), cells deficient for (HtrA2/Omi KO) or reconstituted with WT HtrA2/Omi (HtrA2/Omi KO * WT)] by immunofluorescence microscopy. HtrA2/Omi is indicated by green fluorescence (top left), mitochondria (red) were stained with MitoTracker Orange (top right), cell nuclei (blue) were stained with Hoechst 33342 (bottom left), and an overlay of all stainings is shown at the bottom right. Scale bars, 10 µm. **C** L929Ts cells or **D** MEF, either WT or HtrA2/Omi KO or HtrA2/Omi KO * WT were left untreated or treated for 15 min with 0.5 mM of the parthanatos-inducing agent MNNG. Cells were further incubated with fresh medium without MNNG for 16 h before loss of membrane integrity was determined as a marker for cell death by flow cytometric analysis of PI-positive cells. Each measurement represents the mean of nine determinations, error bars indicate the corresponding SD. ***p < 0.001 (two-tailed unpaired Student’s *t* test). **E**, **F** In parallel, micrographs were generated that depict the effects of parthanatos induction on the morphology of L929 cells and MEF. Seams visible in some of the panels result from the automatic image stitching function of the NYONE cell imager employed in this experiment. Scale bars, 100 µM

In an initial assessment of the role of HtrA2/Omi in parthanatos, we employed L929Ts fibrosarcoma cells, a cell system that we had successfully used in previous analyses of the molecular differences between parthanatos and necroptosis [44]. We deleted HtrA2/Omi in L929Ts cells using the CRISPR/Cas9 system (Fig. 1A, B) and, by measuring loss of membrane integrity as a marker for cell death, found that treatment with 1-methyl-3-nitro-1-nitrosoguanidine (MNNG), an established inducer of parthanatos [42, 44, 58] resulted in the death of parental L929Ts cells, whereas HtrA2/Omi-deficient L929Ts cells were significantly protected (Fig. 1C). To corroborate these results in an independent system, we stimulated embryonic fibroblasts (MEF) from wild-type (WT) and HtrA2/Omi knock-out (KO) mice. Again, a marked cytotoxic response was seen in WT MEF, whereas HtrA2/Omi KO MEF were significantly protected from parthanatos (Fig. 1D).

To further evaluate whether these effects were specific for HtrA2/Omi, we reconstituted HtrA2/Omi KO L929Ts cells and MEF with an expression plasmid for the WT form of HtrA2/Omi (HtrA2/Omi KO * WT; Fig. 1A, B). Reconstitution of HtrA2/Omi fully restored the sensitivity to treatment with MNNG in both L929 cells and MEF (Fig. 1C, D). Correspondingly, when looking at cell morphologies, cultures of HtrA2/Omi WT and HtrA2/Omi KO * WT L929Ts cells and MEF were clearly more affected by treatment with MNNG than HtrA2/Omi KO cells (Fig. 1E, F). Altogether, these results suggested a novel, previously undescribed role of HtrA2/Omi in parthanatos.

### Function of LONP1, PMPCA and PARL in parthanatos

**Fig. 2:**
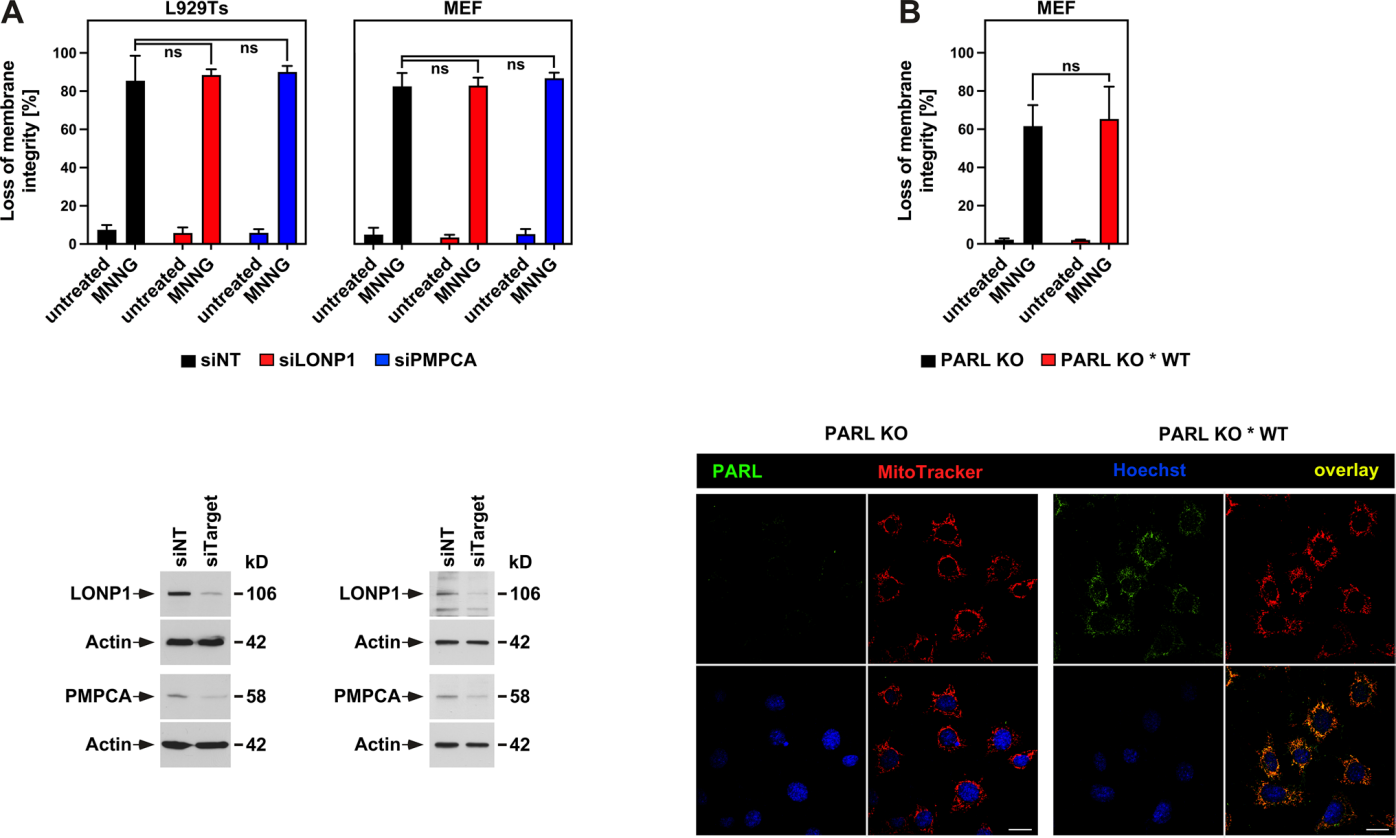
Impact of LONP1, PMPCA and PARL on parthanatos. **A** WT L929Ts cells or MEF were nucleofected with a non-targeting siRNA that does not elicit an RNA interference response (siNT) or with siRNAs specific for murine LONP1 (si LONP1) or PMPCA (siPMPCA). 48 h after nucleofection, the cells were left untreated or stimulated with 0.5 mM MNNG for 15 min and further incubated for 16 h with fresh medium without MNNG before loss of membrane integrity as a marker for cell death was measured by PI staining and flow cytometry (upper panels). Each measurement represents the mean of nine determinations, error bars indicate the corresponding SD. *ns* not significant (two-tailed unpaired Student’s *t* test). Expression of the respective target proteins was analyzed in Western blots for LONP1 and PMPCA. Detection of actin served as control for equal loading (lower panels). **B** PARL-deficient MEF (PARL KO) or PARL-deficient MEF reconstituted with WT FLAG-tagged PARL (PARL KO * WT) were left untreated or stimulated with 0.5 mM MNNG for 15 min and further incubated for 16 h with fresh medium without MNNG before loss of membrane integrity as a marker for cell death was measured by PI staining and flow cytometry (upper panel). Each measurement represents the mean of nine determinations, error bars indicate the corresponding SD. ns, not significant (two-tailed unpaired Student’s *t* test). Lower panel: immunofluorescence analysis to validate the presence of PARL in PARL KO * WT MEF. PARL is indicated by green fluorescence (top left), mitochondria (red) were stained with MitoTracker Orange (top right), cell nuclei (blue) were stained with Hoechst 33342 (bottom left), and an overlay of all stainings is shown at the bottom right. Scale bars, 10 µm

Next, we investigated whether mitochondrial proteases other than HtrA2/Omi were involved in parthanatic cell death. In a screen for proteases that regulate non-apoptotic cell death, we had previously identified Lon peptidase 1, mitochondrial (LONP1) and peptidase, mitochondrial processing subunit alpha (PMPCA) as candidates. We, therefore, targeted LONP1 and PMPCA by RNA interference. As shown in Fig. 2A, downregulation of neither LONP1 nor PMPCA in WT L929Ts cells and MEF had any significant effect on the course of MNNG-induced parthanatos. Likewise, parthanatos proceeded without significant differences in MEF deficient for the protease presenilins-associated rhomboid-like protein, mitochondrial (PARL), and in PARL-deficient MEF that had been reconstituted with PARL (Fig. 2B). These results, therefore, indicate that increased resistance to parthanatos is not a generalized cellular response that can be elicited by manipulating any mitochondrial protease, but rather a feature specifically caused by loss of HtrA2/Omi.

### HtrA2/Omi acts downstream of PARP-1

**Fig. 3:**
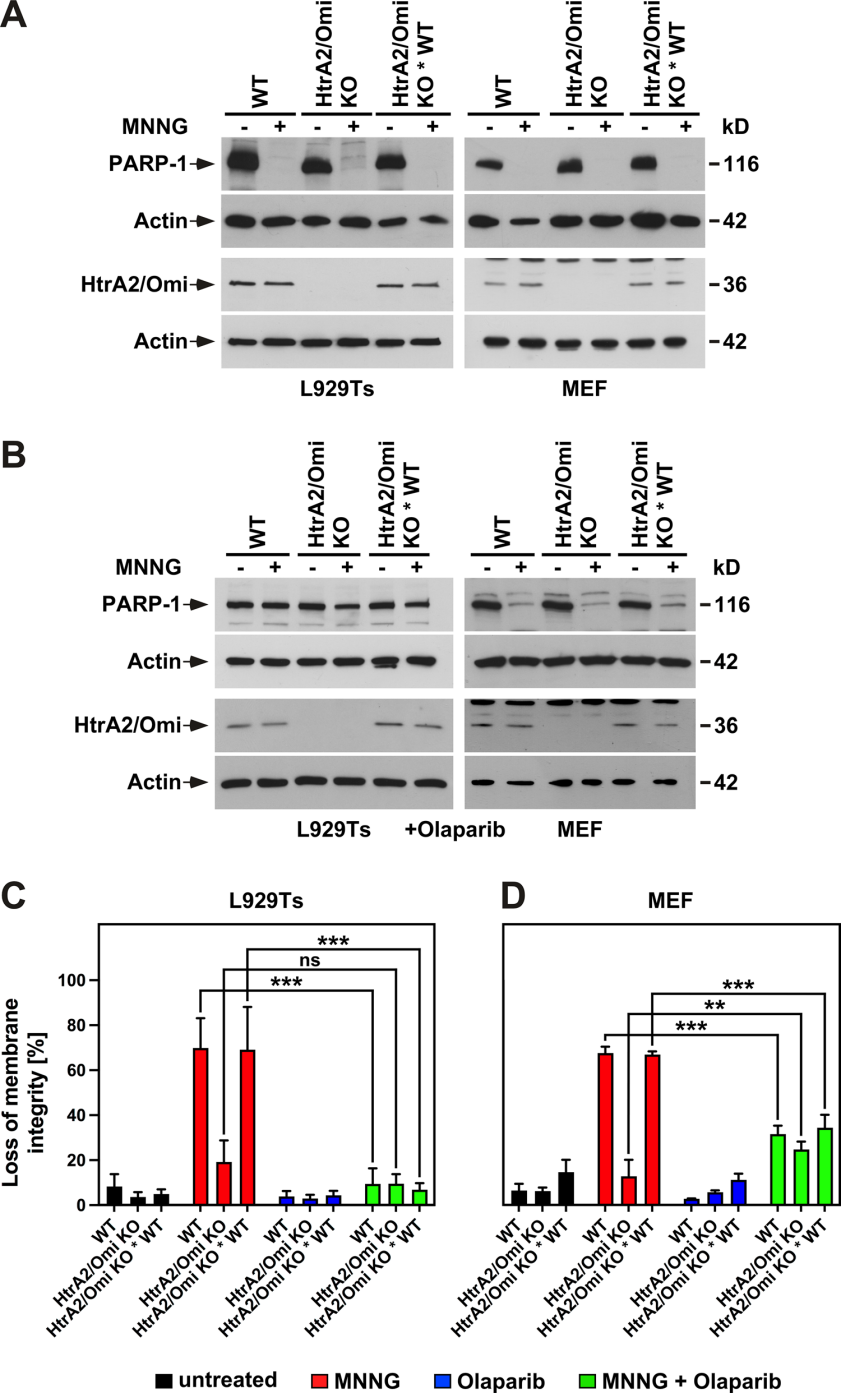
HtrA2/Omi regulates parthanatos downstream of PARP-1. **A** WT, HtrA2/Omi KO or HtrA2/Omi KO * WT L929Ts cells or MEF were left untreated (−) or stimulated with MNNG (+) as in Fig. 1C. **B** In a parallel experiment, cells were additionally treated with 1 µM of the PARP-1 inhibitor olaparib (2 h preincubation before and 16 h incubation after MNNG stimulation). Subsequently, expression of PARP-1, or of HtrA2/Omi was detected by immunoblotting. Detection of actin served as control for equal loading. **C**, **D** Cells were left untreated or stimulated with 0.5 mM MNNG for 15 min and further incubated for 16 h with fresh medium without MNNG in the presence or absence of 1 µM olaparib before loss of membrane integrity as a marker for cell death was determined by flow cytometry and PI staining. Each measurement represents the mean of nine determinations, error bars indicate the corresponding SD. *ns* not significant, **p < 0.01, ***p < 0.001 (Mann–Whitney test)

Subsequently, we addressed the question whether HtrA2/Omi exerted its effects by affecting parthanatos induction or rather by acting as a downstream component of the parthanatic signaling cascade. Hyperactivation of PARP-1 represents the very first step of this cascade and we have previously shown that this process is accompanied by heavy PARP-1 autoPARylation, rendering PARP-1 inaccessible for detection by PARP-1 antibodies and leading to the disappearance of the PARP-1 signal in Western blots [44]. As shown in Fig. 3A, induction of parthanatos by MNNG uniformly led to the disappearance of the full-length PARP-1 signal in WT, HtrA2/Omi KO and HtrA2/Omi KO * WT L929Ts cells and MEF, regardless of the presence or absence of HtrA2/Omi. This clearly shows that the knock-out of HtrA2/Omi does not impact on the levels and activation of PARP-1, and indicates that HtrA2/Omi does not act as a regulator of parthanatos upstream of PARP-1 but rather constitutes a downstream component of the signaling chain, in line with its localization within mitochondria, which relay the parthanatic signal downstream of PARP-1. Accordingly, blockade of the parthanatic signaling cascade by olaparib, an inhibitor of PARP-1, completely prevented the disappearance of the full-length PARP-1 signal in L929 Ts cells (Fig. 3B) and protected them from the cytotoxic effects of MNNG (Fig. 3C) independent from whether HtrA2/Omi was present or absent. In MEF, the effect of olaparib on the disappearance of PARP-1 as well as the protection from MNNG cytotoxicity was not as prominent as in L929Ts cells, but again not affected by the presence or absence of HtrA2/Omi (Fig. 3B, D). As an additional result, the expression levels of HtrA2/Omi were unaltered by treatment of cells with MNNG and/or olaparib (Fig. 3A, B).

### Regulation of parthanatos by HtrA2/Omi occurs by IAP-independent mechanisms

**Fig. 4:**
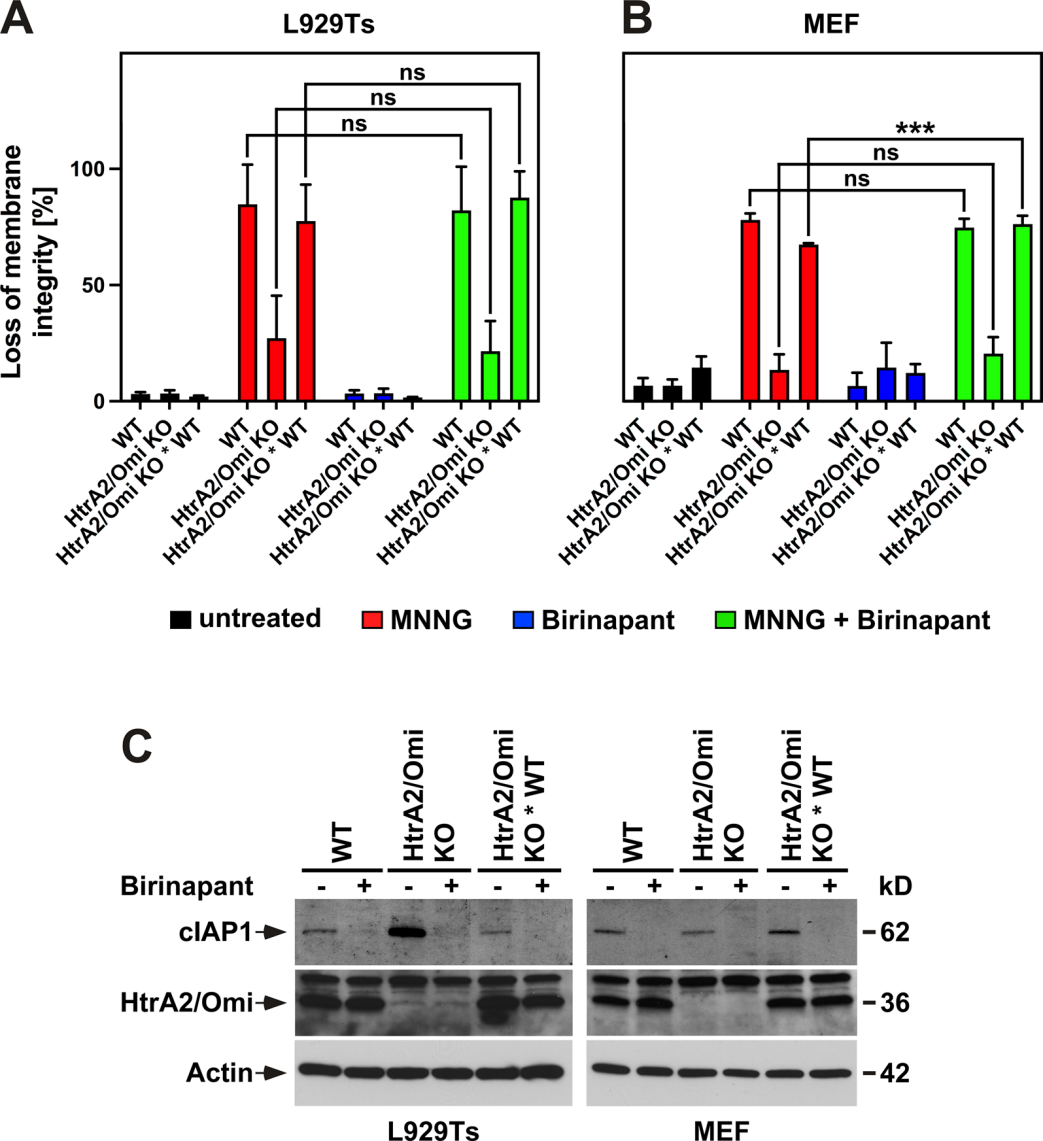
HtrA2/Omi regulates parthanatos by IAP-independent mechanisms. **A** WT, HtrA2/Omi KO or HtrA2/Omi KO * WT L929Ts cells or MEF **B** were left untreated or stimulated with 0.5 mM MNNG for 15 min and further incubated for 16 h with fresh medium without MNNG in the presence (30 min preincubation before and 16 h incubation after MNNG stimulation) or absence of the Smac mimetic birinapant (1 µM). Subsequently, loss of membrane integrity as a marker for cell death was determined by flow cytometry and PI staining. Each measurement represents the mean of nine determinations, error bars indicate the corresponding SD. *ns* not significant, ***p < 0.001 (two-tailed unpaired Student’s *t* test). **C** Cells were left untreated (−) or treated with 1 µM birinapant for 30 min (+) before expression of cIAP1 and HtrA2/Omi was detected by immunoblotting. Detection of actin served as control for equal loading

During apoptosis, HtrA2/Omi binds and cleaves IAP proteins, abolishing their inhibitory effect on caspases and thus enhancing the cell death response. To clarify whether the degradation of IAPs by HtrA2/Omi is also a relevant mechanism in parthanatos, we employed birinapant, a member of the family of Smac mimetics that induce the rapid degradation of IAPs [59]. We reasoned that, if the protection against parthanatos in HtrA2/Omi KO cells was due to a lack of IAP degradation, the addition of birinapant should override this protection by inducing IAP degradation in an HtrA2/Omi-independent manner. As shown in Fig. 4A, B, and contrary to this assumption, the parthanatic resistance of HtrA2/Omi KO L929Ts cells and MEF was completely unaffected by treatment with birinapant. We validated that birinapant was indeed active, showing that treatment with birinapant led to the complete degradation of cIAP1 in WT, HtrA2/Omi KO and HtrA2/Omi KO * WT L929Ts cells and MEF (Fig. 4C), in summary suggesting that HtrA2/Omi regulates parthanatos by IAP-independent mechanisms.

### Function of known and candidate HtrA2/Omi substrates as downstream mediators of parthanatos

**Fig. 5:**
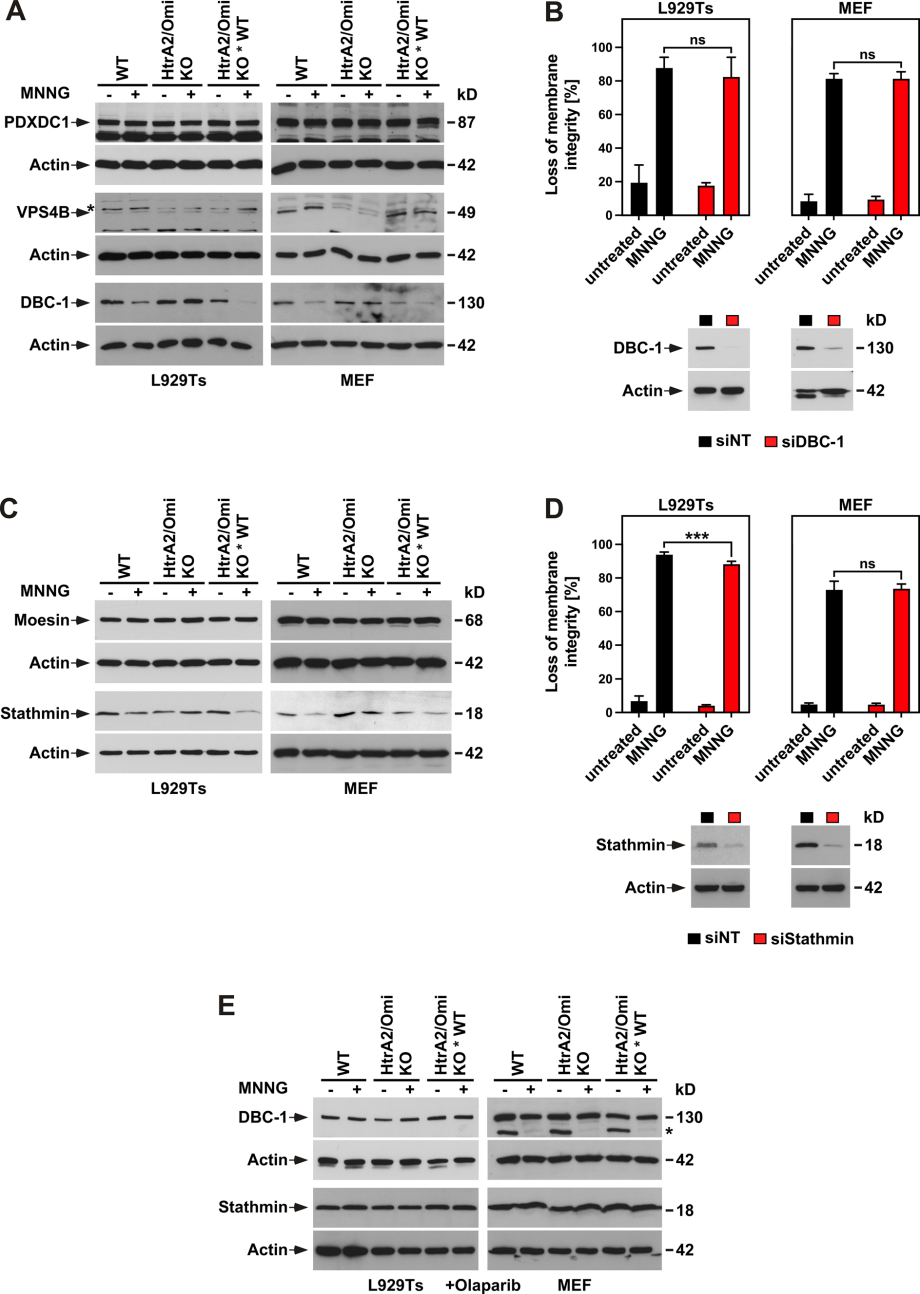
Role of known and candidate HtrA2/Omi substrates in parthanatos. **A** WT, HtrA2/Omi KO or HtrA2/Omi KO * WT L929Ts cells or MEF were left untreated or stimulated with 0.5 mM MNNG for 15 min and further incubated for 16 h with fresh medium without MNNG. Subsequently, cells were analyzed for the presence of PDXDC1, VPS4B and DBC-1 by immunoblotting. For VPS4B, its predicted molecular mass corresponds to the lower of the two bands. The upper, non-specific band is indicated by an asterisk. Detection of actin served as control for equal loading. **B** WT L929Ts cells or MEF were nucleofected with a non-targeting siRNA that does not elicit an RNA interference response (siNT) or with an siRNA specific for murine DBC-1 (siDBC-1). 48 h after nucleofection, the cells were left untreated or stimulated with 0.5 mM MNNG for 15 min and further incubated for 16 h with fresh medium without MNNG before loss of membrane integrity as a marker for cell death was measured by PI staining and flow cytometry (upper panels). Each measurement represents the mean of nine determinations, error bars indicate the corresponding SD. *ns* not significant (two-tailed unpaired Student’s *t* test). In parallel, Western blots for DBC-1 were performed to interrogate its expression. Detection of actin served as control for equal loading (lower panels). **C** WT, HtrA2/Omi KO or HtrA2/Omi KO * WT L929Ts cells or MEF were treated as in **A** and analyzed for the presence of moesin and stathmin by immunoblotting. Detection of actin served as control for equal loading. **D** WT L929Ts cells or MEF were nucleofected with siNT or with an siRNA specific for murine stathmin (siStathmin) and further analyzed as in **B** (upper panels). Each measurement represents the mean of nine determinations, error bars indicate the corresponding SD. *ns* not significant, ***p < 0.001 (two-tailed unpaired Student’s *t* test). Downregulation of stathmin was monitored in Western blots, detection of actin served as control for equal loading (lower panels). **E** WT, HtrA2/Omi KO or HtrA2/Omi KO * WT L929Ts cells or MEF were left untreated or stimulated with 0.5 mM MNNG for 15 min and further incubated for 16 h with fresh medium without MNNG in the presence of the PARP-1 inhibitor olaparib (1 µM; 2 h preincubation before and 16 h incubation after MNNG stimulation). Subsequently, expression of DBC-1, or of Stathmin was detected by immunoblotting. Detection of actin served as control for equal loading. The actin blot for DBC-1 from MEF is identical to the one shown for PARP-1 in Fig. 3B, as all three proteins were analyzed using the same Western blot membrane. This also explains the reappearing signal for PARP-1 (indicated by an asterisk) below DBC-1

In a proteome-wide screen, Vande Walle and coworkers had previously identified pyridoxal dependent decarboxylase domain-containing protein 1 (PDXDC1), vacuolar protein sorting 4 homolog B (VPS4B) and deleted in breast cancer 1 (DBC-1) as substrates of HtrA2/Omi [60]. To evaluate whether the three proteins could represent potential parthanatic downstream mediators of HtrA2/Omi, we assessed whether they were cleaved by HtrA2/Omi after induction of parthanatos. For PDXDC1 and VPS4B, the levels of the uncleaved full-length proteins remained unchanged after treatment of WT, HtrA2/Omi KO and HtrA2/Omi KO * WT L929Ts cells and MEF with MNNG (Fig. 5A), arguing that the two proteins are not parthanatic substrates of HtrA2/Omi. In contrast, the levels of DBC-1 were reduced in MNNG-treated HtrA2/Omi-expressing L929Ts cells and MEF, whereas no such change was detectable in cells deficient for HtrA2/Omi (Fig. 5A). To further elucidate whether DBC-1 might act as a mediator of parthanatos downstream of HtrA2/Omi, we downregulated DBC-1 and assessed its effect on parthanatic cell death. As shown in Fig. 5B, interference with the expression of DBC-1 did not reduce parthanatos in either WT L929Ts cells nor MEF. Also, it did not enhance MNNG-induced cell death, an effect to be expected if DBC-1 needs to be cleaved and inactivated to facilitate parthanatos downstream of HtrA2/Omi. However, the already high levels of parthanatos in MNNG-treated cells might have obscured the detection of such an enhancing effect, leaving the possibility that DBC-1 may represent a parthanatic downstream mediator of HtrA2/Omi.

We also analyzed the proteins moesin and stathmin which we had previously identified in a screen for proteins cleaved during non-apoptotic cell death. As shown in Fig. 5C, the induction of parthanatos did not change the levels of uncleaved full-length moesin in any cell line, arguing against moesin being a parthanatic substrate of HtrA2/Omi. For stathmin, a slight decrease of the full-length protein was detectable in MNNG-treated WT and HtrA2/Omi KO * WT L929Ts cells and MEF expressing HtrA2/Omi, but not in HtrA2/Omi KO cells (Fig. 5C), indicating that stathmin could be degraded during parthanatos in a HtrA2/Omi-dependent manner. Yet, when we analyzed the effect of stathmin downregulation on parthanatic cell death, no effect at all was detectable in WT MEF, whereas a minimal protection from parthanatos instead of an enhancement was visible in WT L929Ts cells (Fig. 5D). Similar to DBC-1, the high degree of cell death already present upon MNNG-treatment might have interfered with the detection of such an enhancement, and thus, stathmin may likewise represent a parthanatic downstream mediator of HtrA2/Omi.

Notwithstanding, pharmacological inhibition of the parthanatos-initiating enzyme PARP-1 with olaparib prevented the cleavage of both DBC-1 and stathmin (Fig. 5E), demonstrating that their cleavage is a specific feature of parthanatic cell death.

### HtrA2/Omi does not exit mitochondria during parthanatos

**Fig. 6:**
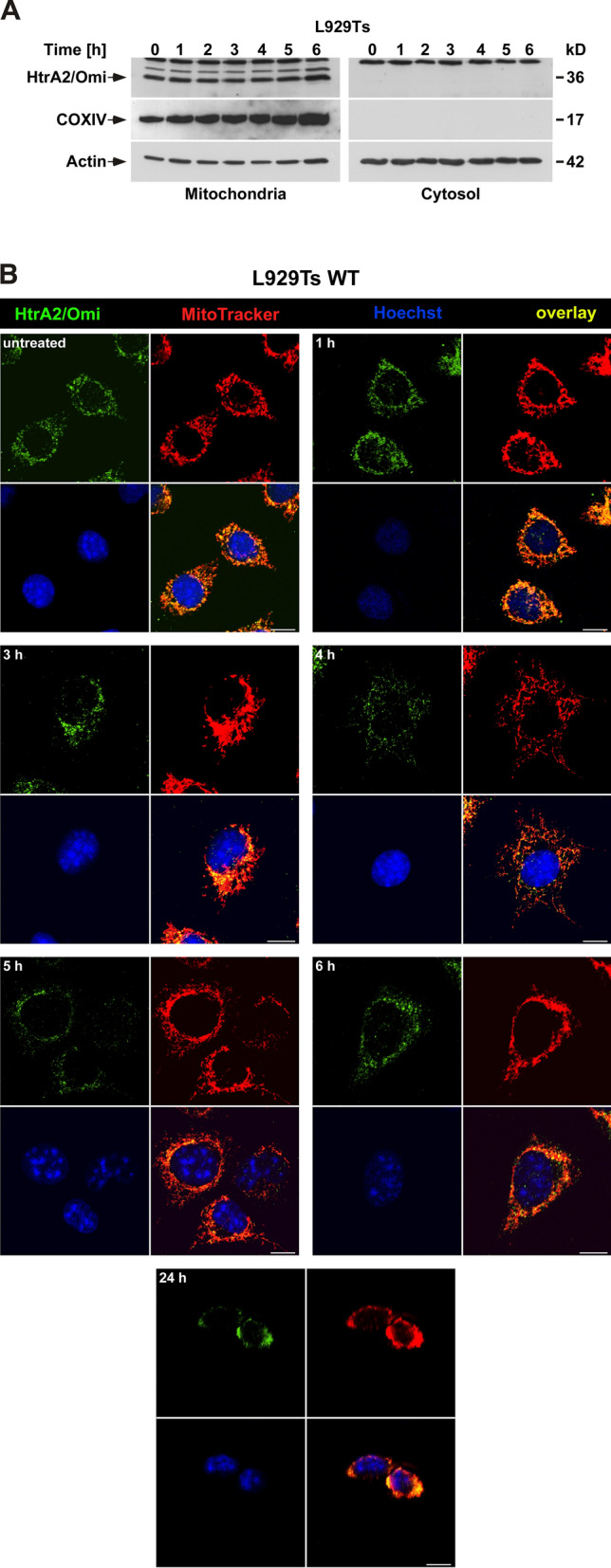
HtrA2/Omi is not released from mitochondria into the cytosol during parthanatos. **A** WT L929Ts cells were left untreated (0) or treated with 0.5 mM MNNG for 15 min and further incubated with fresh medium without MNNG for the indicated times. Subsequently, mitochondrial and cytosolic fractions were prepared and the presence of HtrA2/Omi in these fractions was examined by Western blot. Detection of the mitochondrial marker COXIV was used to ensure that the cytoplasmic fractions were free from mitochondrial contaminations. Given its tight association with mitochondria [78], detection of actin served as a loading control for both mitochondrial as well as cytosolic fractions. **B** WT L929Ts cells were left untreated or stimulated with 0.5 mM MNNG for 15 min and further incubated for the indicated times with fresh medium without MNNG before they were analyzed by immunofluorescence microscopy. HtrA2/Omi is indicated by green fluorescence (top left), mitochondria (red) were stained with MitoTracker Orange (top right), cell nuclei (blue) were stained with Hoechst 33342 (bottom left), and an overlay of all stainings is shown at the bottom right. Scale bars, 10 µm

For apoptosis, it is well established that HtrA2/Omi is released from mitochondria into the cytosol where it enhances the death response [1–4, 21–28]. For necroptosis, conflicting studies either suggest that HtrA2/Omi is retained within the mitochondria [1, 29] or that it is released into the cytoplasm [30, 35]. For parthanatos, to the best of our knowledge, this issue has not been addressed at all. Therefore, we induced parthanatos in WT L929Ts cells and MEF in a time course experiment and analyzed mitochondrial and cytosolic fractions by Western blot. In both cell lines, the HtrA2/Omi content of mitochondria did not decrease after parthanatos induction and no HtrA2/Omi became detectable in the cytosolic fractions over the entire timecourse (Fig. 6A, Supplementary Fig. 1B). To further corroborate these findings, we determined the intracellular localization of HtrA2/Omi after parthanatos induction in WT, and additionally in HtrA2/Omi KO * WT L929Ts cells and MEF by immunofluorescence in an extended time course experiment. At all investigated time points, and even in the few surviving cells 24 h after induction of parthanatos, the intramitochondrial localization of HtrA2/Omi remained unaltered (Fig. 6B, Supplementary Fig. 1A, C, D). In contrast, when we induced apoptosis in these cells in a control experiment, immunofluorescence analyses revealed that HtrA2/Omi became clearly detectable outside mitochondria within 3 h after induction of apoptosis. Noteworthy, from a time point of 6 h, we found that HtrA2/Omi primarily colocalized with mitochondria again (Supplementary Fig. 2), indicating that the mitochondrial release of HtrA2/Omi during apoptosis might be transient. Altogether, these results support the notion that unlike in apoptosis, in parthanatos, HtrA2/Omi does not need to exit the mitochondria to exert its cell death-promoting functions.

### Mass-spectrometric screen for potential downstream mediators of HtrA2/Omi by N‑terminomics (HYdrophobic Tagging-Assisted N-termini Enrichment, HYTANE)

**Fig. 7:**
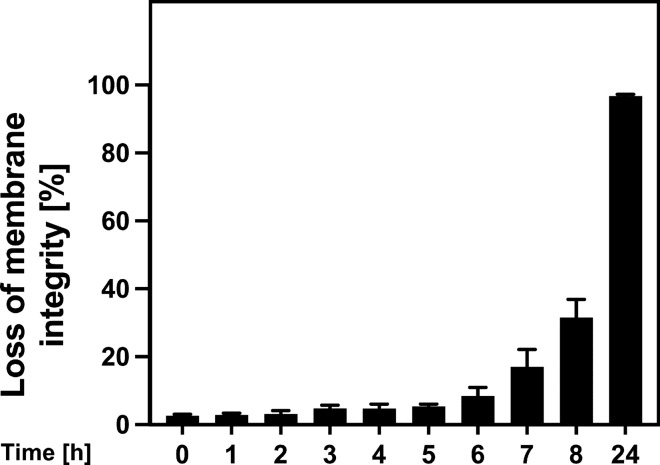
Timecourse of parthanatos in WT MEF. Cells were left untreated or stimulated with 0.5 mM MNNG for 15 min and further incubated for the indicated times. Subsequently, loss of membrane integrity as a marker for cell death was determined by flow cytometry and PI staining. Each measurement represents the mean of nine determinations, error bars indicate the corresponding SD

**Fig. 8:**
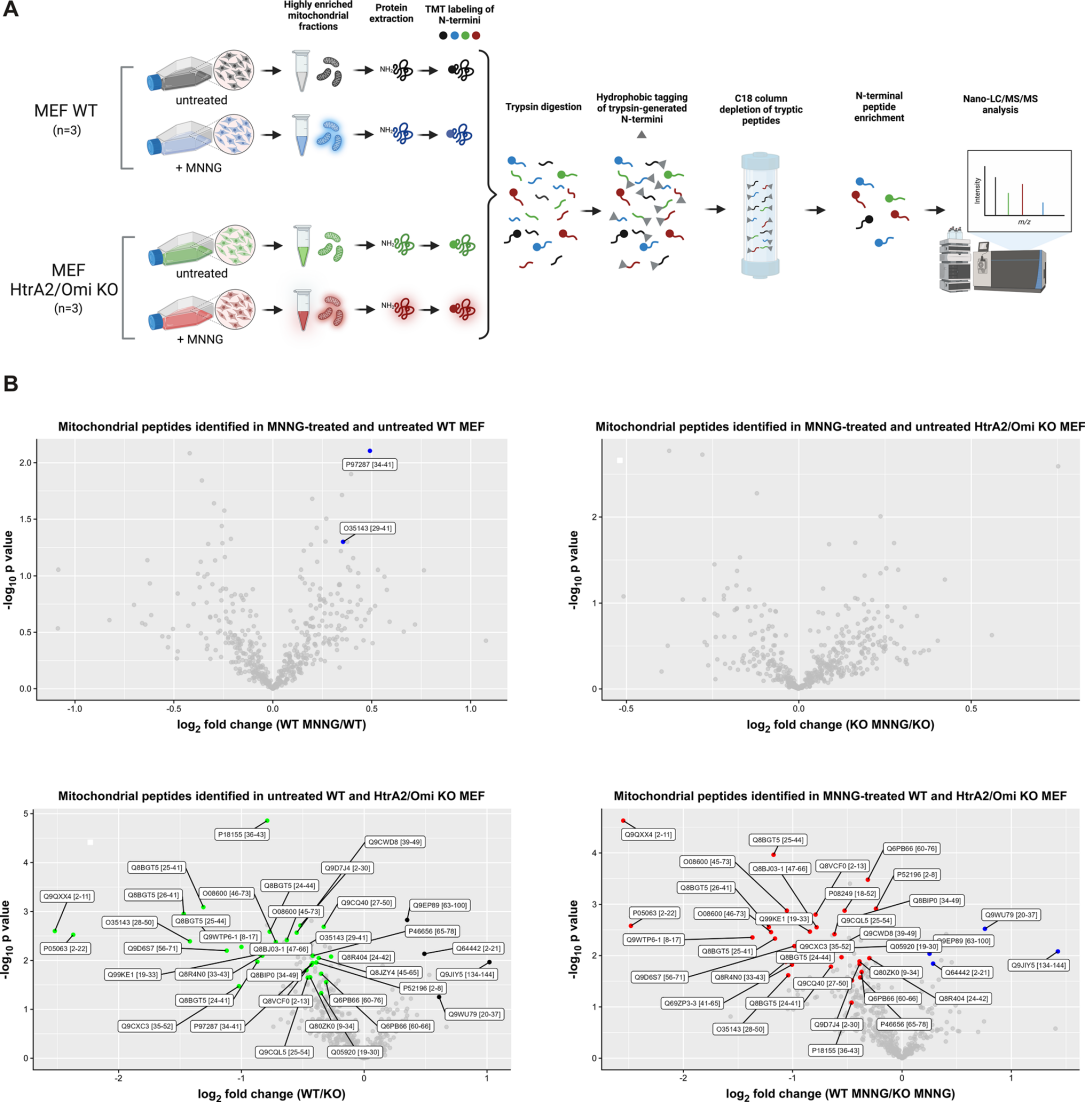
Mass-spectrometric HYTANE screen for potential downstream mediators of HtrA2/Omi. **A** Schematic workflow of the HYTANE experiment to enrich and identify peptides with protease-generated neo-N-termini in untreated and parthanatic WT and HtrA2/Omi KO MEF. See "Materials and methods" for a complete description of the experimental details. Figure created with BioRender.com. **B** Volcano plots of mitochondrial peptides identified by HYTANE in untreated and MNNG-treated WT and HtrA2/Omi KO MEF. Normalized, scaled abundance ratios (to 1) were log_2_-transformed and subjected to statistical analysis (ANOVA, using 5% FDR). A post-hoc Tukey (HSD) test 0.05 (5%) was implemented to determine the significant pairs in the ANOVA. Peptides with statistically significant differences in their abundance between conditions are shown in color (following the color code given in **A**), together with their master protein accession and the position of the peptide within the master protein. The color of the peptides in each panel indicates the condition in which the peptide was more abundant. Grey dots indicate peptides where differences in the protein abundance between the two sample conditions did not reach statistical significance

Since all of the potential downstream mediators of HtrA2/Omi that we had tested so far had turned out to be non-essential for parthanatos, and since HtrA2/Omi obviously exerted its death-promoting functions inside the mitochondria, we performed a mass-spectrometric screen for mitochondrial proteins that are cleaved by HtrA2/Omi in parthanatic cells and which, therefore, might relay the death signal downstream of HtrA2/Omi. For this purpose, WT and HtrA2/Omi KO MEF were treated with MNNG and further cultivated for 4 h, a timeframe short enough to avoid parthanatic disintegration of cells (Fig. 7), but long enough to allow HtrA2/Omi-dependent cleavage of intramitochondrial proteins. Thereafter, highly enriched mitochondrial fractions from untreated or MNNG-stimulated WT and HtrA2/Omi KO MEF were analyzed by HYTANE, a technique that can identify and distinguish protease-generated neo-N termini from the natural N termini of mature proteins [61] (Fig. 8A), and also allows to detect differences in protein abundance among samples. Notably, when we compared the protein abundance ratios between MNNG-treated and untreated WT MEF, only one of the identified 252 mitochondrial proteins (Supplementary Table 1) showed statistically significant differences (coiled-coil-helix-coiled-coil-helix domain-containing protein 1; Table 1), and the analysis of MNNG-treated vs. untreated HtrA2/Omi KO MEF did not reveal any candidates with statistically significant changes in their abundance (Table 1). Consistently, a comparison between untreated WT and HtrA2/Omi KO MEF identified only four mitochondrial proteins with statistically significant changes in abundance (Table 1). In WT vs. HtrA2/Omi KO MEF that had been treated with MNNG, the same proteins were identified, although differences in the protein abundance did not reach statistical significance for one protein (N(G),N(G)-dimethylarginine dimethylaminohydrolase 2; Table 1). Therefore, we re-analyzed the data using a statistically less stringent approach by pairwise comparing groups using Student’s *t* test (and additionally by a modified ANOVA) as outlined in "Materials and methods" (Supplementary Table 2). Notably, a comparison between MNNG-treated and untreated WT MEF by *t* test did not identify any protein with significant abundance changes (Table 2), and the same analysis in HtrA2/Omi KO MEF revealed only one protein, however, not reaching significance in the modified ANOVA (Table 2). When comparing untreated WT with HtrA2/Omi KO MEF via *t* test, again only four mitochondrial proteins showed statistically significant differences in their protein abundance (three of them being identical to those identified in the first analysis), and only two of them (alanine aminotransferase 2, ribosome-recycling factor, mitochondrial) also showed statistically significant differences in the modified ANOVA (Table 2). When we compared MNNG-treated WT with HtrA2/Omi KO MEF, eight proteins with statistically significant differences were identified by *t* test. Three of these eight proteins (alanine aminotransferase 2, ribosome-recycling factor, mitochondrial, and thiosulfate sulfurtransferase) had already been identified in the *t* test analysis of untreated WT and HtrA2/Omi KO MEF, and six of the eight proteins failed to show significant differences in the modified ANOVA (Table 2). In summary, these results suggest that the absence of HtrA2/Omi by itself does not result in major alterations of the mitochondrial proteome and that the induction of parthanatos in HtrA2/Omi-proficient and -deficient MEF does not substantially alter the abundance of their mitochondrial proteins.

**Table 1:**
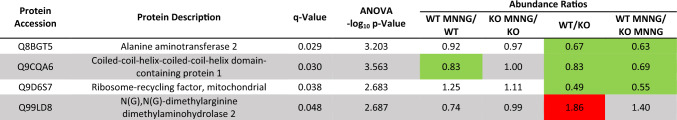
Mitochondrial proteins with statistically significant differences in their abundance in untreated and MNNG-treated WT vs. HtrA2/Omi KO MEF [ANOVA of log_2_-transformed scaled abundances using 5% FDR and post-hoc Tukey’s HSD test (5%)]

Green indicates that the protein is more abundant in the denominator while red signifies that the protein is more abundant in the numerator. No color: differences in the protein abundance between the two sample conditions did not reach statistical significance

**Table 2:** Mitochondrial proteins with statistically significant differences in their abundance in untreated and MNNG-treated WT vs. HtrA2/Omi KO MEF [ANOVA of scaled abundances using 5% FDR and post-hoc Tukey’s HSD test (5%), and additional pairwise comparison of groups by Student’s *t* test]

Protein accession	Protein description	*q *value	ANOVA − log_10_ *p *value	ANOVA significant?	*t* test			
					WT MNNG/WT	KO MNNG/KO	WT/KO	WT MNNG/ KO MNNG
Q8BGT5	Alanine aminotransferase 2	0.049	3.365	Yes			0.005	0.006
Q9D6S7	Ribosome-recycling factor, mitochondrial	0.053	3.001	Yes			0.006	0.013
P52196	Thiosulfate sulfurtransferase	0.074	2.533	No			0.012	0.017
Q99LD8	N(G),N(G)-dimethylarginine dimethylaminohydrolase 2	0.092	2.337	No			0.025	
O35680	28S ribosomal protein S12, mitochondrial	0.122	2.404	No				0.014
P58059	28S ribosomal protein S21, mitochondrial	0.176	2.061	No				0.014
Q8R4N0	Citrate lyase subunit beta-like protein, mitochondrial	0.198	1.696	No				0.017
Q8K411-1	Presequence protease, mitochondrial	0.251	1.557	No				0.035
Q99LY9	NADH dehydrogenase [ubiquinone] iron-sulfur protein 5	0.306	1.247	No				0.037
P02340	Cellular tumor antigen p53	0.805	0.314	No		0.039		

Empty cells: differences in the protein abundance between the two sample conditions did not reach statistical significance

As the primary goal of the HYTANE experiment, we next analyzed the data with regard to protease-generated neo-N termini in the samples. A total of 470 mitochondrial peptides were identified, representing 282 master proteins (Supplementary Table 3). 261 peptides had an N terminus identical to that of the mature protein or located within the transit-/signal-/or pro-peptide of the unprocessed pro-protein (Supplementary Table 3). For further 25 peptides, the exact size of the transit peptide of these proteins (and thus the exact location of the N terminus of the processed mature protein) is not known (https://beta.uniprot.org/), i.e., it could not be determined whether their N termini correspond to the natural N termini of the respective mature proteins (Supplementary Table 3). However, the N termini of these 25 peptides were all located between amino acid positions 2 and 91 of the unprocessed pro-protein and thus may represent the N termini of partially or fully processed proteins rather than internal cleavage sites. The remaining 184 peptides contained internal N termini not identical to those of the mature master proteins (Supplementary Table 3). However, for 87 of these peptides, their N termini were located within the first 10, and for another 24 within the first 20 amino acids of the corresponding mature proteins (Supplementary Table 3), raising doubts whether they indeed represent genuine protease-generated neo-N termini. 73 peptides had N termini that located between positions 21 and 431 of the respective mature proteins. Yet, only 10 of them (all locating between positions 24 and 47, and representing only six different master proteins) showed statistically significant differences in their peptide abundance ratios between sample conditions. Moreover, except for one single peptide (corresponding to the master protein induced myeloid leukemia cell differentiation protein Mcl-1 homolog), none of these few differences were due to treatment of the cells with MNNG, but rather became apparent when comparing WT with HtrA2/Omi KO MEF (Supplementary Table 3, Table 3, Fig. 8B). When we alternatively re-screened all 470 originally identified mitochondrial peptides exclusively for statistically significant differences in their abundance ratios between sample conditions, in addition to these 10 peptides, we identified 29 other peptides (Supplementary Table 3, Table 3, Fig. 8B). Yet, 18 of them represented the N termini of their mature proteins, for two, the N terminus of the mature protein is unknown (their N termini locate at amino acid positions 20 and 25 of the unprocessed pro-protein, again likely representing the N termini of partially or fully processed proteins rather than internal cleavage sites), and the N termini of the remaining nine peptides located within the first 13 amino acids of their mature proteins, thus possibly not resulting from protease-mediated cleavage.

**Table 3:**
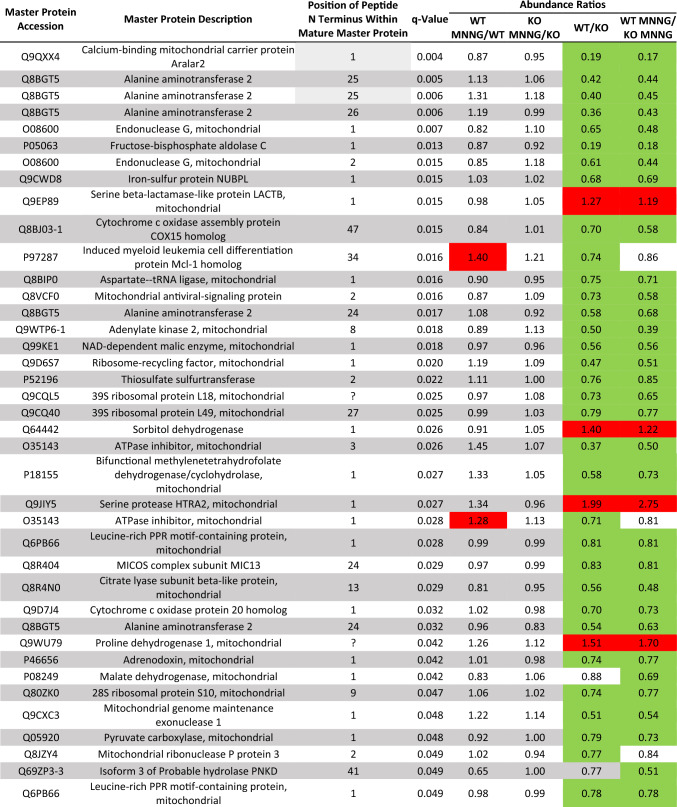
Mitochondrial peptides with statistically significant differences in their abundance in untreated and MNNG-treated WT vs. HtrA2/Omi KO MEF [ANOVA of log_2_-transformed scaled abundances using 5% FDR and post-hoc Tukey’s HSD test (5%)]

Green indicates that the protein is more abundant in the denominator while red signifies that the protein is more abundant in the numerator. No color: differences in the protein abundance between the two sample conditions did not reach statistical significance. "?" indicates that the N terminus of the mature master protein is unknown

As above for the protein data, we performed a statistically less stringent re-analysis of the peptide data (i.e., by comparing groups using Student’s *t* test and additionally a modified ANOVA), and furthermore filtered for peptides with an N terminus located within the corresponding mature master protein. In this approach, we identified a total of 14 peptides with statistically significant abundance differences between sample conditions (Supplementary Table 4, Table 4). Once more, all of these differences were observed when we compared WT with HtrA2/Omi KO MEF, but none of them resulted from treatment of the cells with MNNG (Table 4). Altogether, these results suggested that cleavage of proteins by HtrA2/Omi (or by other proteases) may not be a key requirement for the execution of parthanatic cell death.

**Table 4:** Mitochondrial peptides with statistically significant differences in their abundance in untreated and MNNG-treated WT vs. HtrA2/Omi KO MEF [ANOVA of scaled abundances using 5% FDR and post-hoc Tukey’s HSD test (5%), and additional pairwise comparison of groups by Student’s *t* test]

Master protein accession	Master protein description	Position of peptide N terminus within mature master protein	*q* value	ANOVA significant?	*t* test			
					WT MNNG/WT	KO MNNG/KO	WT/KO	WT MNNG/KO MNNG
Q8BGT5	Alanine aminotransferase 2	25	0.003	Yes			0.00	0.01
Q8BGT5	Alanine aminotransferase 2	25	0.002	Yes			0.00	0.00
Q8BGT5	Alanine aminotransferase 2	26	0.006	Yes			0.01	0.01
O55125	Protein NipSnap homolog 1	36	0.023	Yes			0.01	0.02
Q9WTP6-1	Adenylate kinase 2, mitochondrial	8	0.004	Yes			0.01	0.00
O35143	ATPase inhibitor, mitochondrial	3	0.013	Yes			0.01	0.02
Q8R4N0	Citrate lyase subunit beta-like protein, mitochondrial	13	0.018	Yes			0.01	0.01
Q8BGT5	Alanine aminotransferase 2	24	0.037	Yes			0.02	
O08600	Endonuclease G, mitochondrial	2	0.002	Yes				0.00
Q69ZP3-3	Isoform 3 of Probable hydrolase PNKD	41	0.030	Yes				0.01
Q9Z110-1	Delta-1-pyrroline-5-carboxylate synthase	40	0.034	Yes				0.02
Q8BTW8	CDK5 regulatory subunit-associated protein 1	2	0.013	Yes				0.02
Q8K411-1	Presequence protease, mitochondrial	14	0.073	No				0.05
Q8VCX5-2	Isoform 2 of calcium uptake protein 1, mitochondrial	25	0.082	No				0.03

Empty cells: differences in the protein abundance between the two sample conditions did not reach statistical significance

### The protease function of HtrA2/Omi is dispensable for parthanatos

**Fig. 9:**
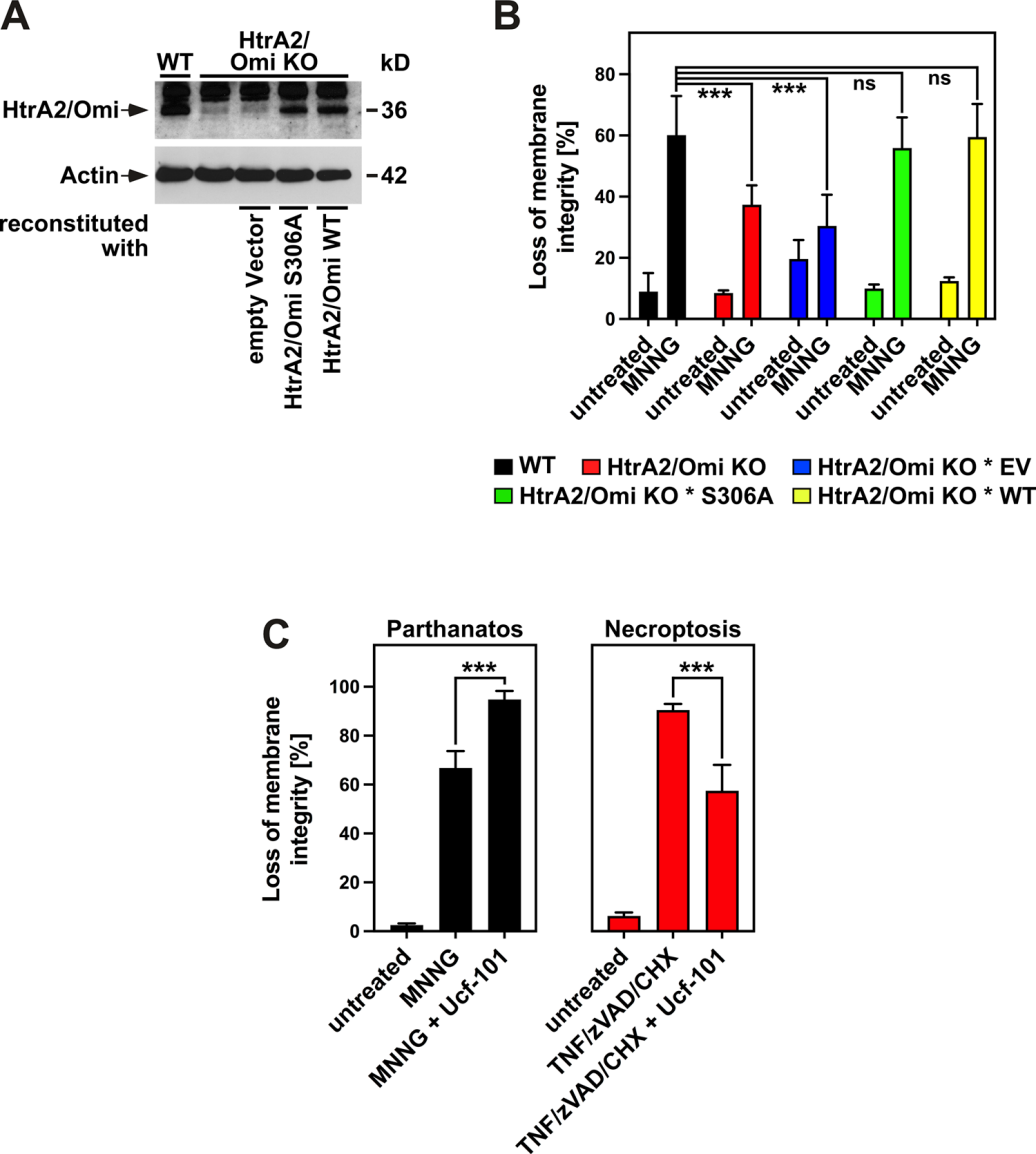
HtrA2/Omi governs parthanatos independent from its protease activity. **A** Western blot to validate the presence or absence of HtrA2/Omi in WT and HtrA2/Omi KO MEF, and in HtrA2/Omi KO MEF reconstituted with the protease-inactive S306A mutant of HtrA2/Omi. Cells reconstituted with empty vector or with WT HtrA2/Omi were originally included in this analysis and are shown for completeness. Detection of actin served as control for equal loading. **B** WT and HtrA2/Omi KO MEF, together with HtrA2/Omi KO MEF reconstituted with empty vector (HtrA2/Omi KO * EV) and HtrA2/Omi KO * S306A and HtrA2/Omi KO * WT MEF were left untreated or stimulated with 0.5 mM MNNG for 15 min and further incubated for 16 h with fresh medium without MNNG before loss of membrane integrity was determined by flow cytometry and PI staining as a marker for cell death. Each measurement represents the mean of nine determinations, error bars indicate the corresponding SD. *ns* not significant, ***p < 0.001 (two-tailed unpaired Student’s *t* test). **C** Parental L929Ts cells were left untreated or stimulated with 1 mM MNNG as in **B** or pretreated with 50 µM Ucf-101 for 2 h and then stimulated with 1 mM MNNG as in **B**, with 50 µM Ucf-101 present during the 16 h incubation period (left panel). Separately, cells were left untreated or, in the absence or presence of 50 µM Ucf-101, pretreated with 20 µM zVAD-fmk and 1 µg/ml CHX for 2 h and further incubated for 5 h after addition of 100 ng/ml TNF to induce necroptosis (right panel). Subsequently, loss of membrane integrity was determined by flow cytometry and PI staining as a marker for cell death. Each measurement represents the mean of nine determinations, error bars indicate the corresponding SD. ***p < 0.001 (two-tailed unpaired Student’s *t* test)

Given that the function of HtrA2/Omi in parthanatos did neither require its exit into the cytoplasm nor the cytoplasmic proteolysis of IAPs or of other known or candidate substrates of HtrA2/Omi, and given that the mass-spectrometric HYTANE analyses had not provided evidence that intramitochondrial cleavage of proteins by HtrA2/Omi is required for parthanatos, we next examined whether parthanatic cell death was dependent on the catalytic activity of HtrA2/Omi at all. For this purpose, we reconstituted HtrA2/Omi KO MEF with an expression plasmid coding for the protease-inactive S306A mutant of HtrA2/Omi [62] (Fig. 9A). Remarkably, reconstitution with HtrA2/Omi S306A rendered HtrA2/Omi KO * S306A MEF as sensitive to parthanatos as reconstitution with the proteolytically active form of HtrA2/Omi or as WT MEF that naturally express HtrA2/Omi (Fig. 9B). In an independent approach, Ucf-101, an established inhibitor of the enzymatic activity of HtrA2/Omi [63], was completely ineffective in protecting parental L929Ts cells from parthanatos while it simultaneously provided significant protection from necroptosis, identical to previous findings [33] and validating its efficacy (Fig. 9C). Together, these results demonstrate that HtrA2/Omi controls parthanatos independent from its proteolytic activity.

## Discussion

The impact of HtrA2/Omi as a mediator of regulated cell death is well established for apoptosis [1–4, 21–28] and necroptosis [4, 29–34]. Here, we present evidence that HtrA2/Omi represents an important component also in a third modality of regulated cell death, i.e., parthanatos. In our study, we found that the deletion of HtrA2/Omi consistently conferred protection from parthanatos. Yet, it should be pointed out that this protection was not as pronounced as the protection conferred by deletion or downregulation of PARP-1 [44] or after pharmacological inhibition of PARP-1 with olaparib (Fig. 3C). Therefore, HtrA2/Omi most likely represents a modulator of parthanatos rather than a core component such as PARP-1. Our findings also indicate that the role of HtrA2/Omi in parthanatos is mechanistically distinct from that in apoptotic or necroptotic cell death. In apoptosis, HtrA2/Omi translocates from the mitochondria into the cytosol where it proteolytically cleaves multiple substrates such as IAPs and thereby enhances the apoptotic demise of the cell [1–4, 21–28]. In necroptosis, although a cytoplasmic release of HtrA2/Omi is still contentious [1, 29, 30, 35], we [33, 34] and others [35] have provided evidence that the protease activity of HtrA2/Omi is essential for its proper function also in this modality of cell death. In parthanatos, however, our data indicate that HtrA2/Omi neither needs to leave the mitochondria nor that its proteolytic activity is required to control this form of cell death. Given that mitochondria are key components of the parthanatic signaling cascade downstream of PARP-1, the intramitochondrial localization of HtrA2/Omi is also consistent with our finding that HtrA2/Omi exerts its functions downstream of PARP-1. Likewise, the dispensability of HtrA2/Omi’s protease activity fits well with our observation that cytosolic cleavage of IAPs by HtrA2/Omi, as it occurs in apoptosis, is not a mechanism by which HtrA2/Omi regulates parthanatos.

Our results also demonstrate that the effects of HtrA2/Omi on parthanatos are specific for HtrA2/Omi. PARL, a distinct mitochondrial protease that has recently been shown to facilitate apoptosis by cleaving and maturating the proapoptotic protein Smac [64], is obviously not a regulator of parthanatos, as a deletion of PARL had no protective effect on the course of parthanatic cell death. Likewise, compromising the function of LONP1, another mitochondrial protease whose inhibition has been reported to cause the death of lymphoma cells [65], or of PMPCA, a subunit of the mitochondrial processing peptidase (MPP) complex [11] which we had identified in an earlier screen for proteases potentially relevant in non-apoptotic cell death, did not affect parthanatic cell death. Therefore, the function of HtrA2/Omi in parthanatos cannot be replaced by other mitochondrial proteases, a finding also to be expected for and consistent with a protease-independent regulation of parthanatos by HtrA2/Omi.

The concept of a protease-independent function of HtrA2/Omi in parthanatos also explains why we did not observe cleavage of moesin or the known HtrA2/Omi substrates PDXDC1 and VPS4B [60] in parthanatic cells. For DBC-1 as another reported substrate of HtrA2/Omi [60] as well as for stathmin, induction of parthanatos caused a reduction of the full-length proteins in HtrA2/Omi-proficient, but not -deficient cells. Although the downregulation of both proteins did not noticeably enhance parthanatos further, this might have escaped detection due to the already high levels of cell death induced by MNNG in our experimental approach. This issue will have to be clarified in future experiments, e.g., by employing milder conditions that induce parthanatos at reduced levels and which more easily permit detection of a potential enhancement by downregulation of DBC-1 or stathmin. Regardless, our results also suggest that their cleavage, although it is a specific feature of parthanatic cell death, is most likely a secondary event and mediated by proteases that are activated downstream of HtrA2/Omi in the course of the parthanatic demise of the cell.

In addition to our experimental evidence directly demonstrating that the protease activity of HtrA2/Omi is dispensable for its function in parthanatos, this notion is further supported by the results of our mass-spectrometric screen. Although originally intended to identify new proteolytic substrates of HtrA2/Omi cleaved during parthanatos, the data obtained in the screen show that treatment of WT or HtrA2/Omi KO MEF with MNNG to induce parthanatos caused virtually no (i.e., one) statistically significant changes in mitochondrial protein abundance in the respective cell lines. Similarly, when we compared protein abundances between WT and HtrA2/Omi KO MEF, the detected statistically significant differences remained essentially unchanged after treatment of the cells with MNNG. In accordance, in our analysis of protease-generated neo-N termini, i.e., protein cleavage in parthanatic cells, again only one single hit with statistical significance was identified between cells that had been treated with MNNG or not. Although we cannot fully rule out the possibility that some peptides were not detected in our analyses, collectively, the results from the mass-spectrometric screen strongly argue that the induction of parthanatos does not entail a substantial proteolytic cleavage of mitochondrial proteins, in accordance with a protease-independent mode of action of HtrA2/Omi in this modality of regulated cell death.

As a side note, in our stable reconstitution experiments with the protease-inactive HtrA2/Omi S306A mutant, we consistently observed in multiple attempts that the generated HtrA2/Omi KO * S306A MEF rapidly lost expression of the construct. It appears as if there is counterselection against the presence of a protease-inactive HtrA2/Omi protein, and therefore, although the protease activity of HtrA2/Omi seems dispensable for parthanatos, it is likely essential for the overall homeostasis and fitness of a cell. This assumption is corroborated by independent studies showing that disturbances of the proteolytic activity of HtrA2/Omi cause the generation of reactive oxygen species, accumulation of unfolded mitochondrial proteins, dysfunction of the mitochondrial respiration and a loss of mitochondrial competence [14, 66–68].

An obvious question that arises from our results is how HtrA2/Omi modulates parthanatic cell death, if not via its protease activity. The most likely explanation is that HtrA2/Omi acts as an intramitochondrial scaffolding protein. Support for this assumption comes from studies showing that the homologous protease PfHtrA2 similarly fulfils a non-protease/chaperone role in the life cycle of the human malaria parasite *Plasmodium falciparum* [69], that HtrA2/Omi itself prevents the aggregation of amyloid β1-42 in a protease-independent manner by acting as a chaperone protein [16], or that the protease-activity of HtrA2/Omi is not required to induce apoptosis in Apollon/BRUCE-deficient cells [24]. A scaffolding function of HtrA2/Omi appears also plausible given that its C-terminal PDZ domain represents a protein–protein interaction module [7, 8]. Accordingly, multiple binding partners (often also substrates) of HtrA2/Omi have been described, such as DUSP9 [70], XIAP, cIAP1, cIAP2, Apollon/BRUCE [1, 4, 22–24], ARMC8 [71], OPA1 [72], ped/pea-15 [25], WARTS [73], GRIM-19 [74], Presenilin [75], HAX-1 [26], WT1 [27], p73 [28], actin, vimentin, α- and β-tubulin [60], integrin α7 [76], or RIPK1 [35]. Yet, parthanatic interactors of HtrA2/Omi need to be mitochondrial proteins, which limits the above list to OPA1, GRIM-19 and HAX-1, none of which have so far been linked to the parthanatic signaling cascade. In recent work, Botham and colleagues have identified 59 additional mitochondrial interaction partners of HtrA2/Omi in a proximity-dependent biotin identification (BioID) screen for protease proximity partners, and 15 further candidates by immunoprecipitation coupled with mass spectrometry (IP-MS) [12]. Of those, eight (APOOL, CHCHD3, CHCHD6, DNAJC11, IMMT, MTX1, MTX2, SAMM50) are components of the mitochondrial intermembrane space bridging complex and 19 (ACAD9, ATPAF1, COX4I1, COX5A, COX5B, COX6C, COX15, NDUFA2, NDUFA8, NDUFA12, NDUFA13, NDUFS1, NDUFS2, NDUFS8, NDUFV2, NDUV3, SDHA, SELRC1, TTC19) of the electron transport chain. 46 other candidates (ACOT2, AFG3L2, AK2, ATAD3A, ATAD3B, BCL2L13, C1QBP, CCDC58, CKMT1B, CLPB, CPOX, CPS1, EARS2, EFHA1, ENDOG, FAM54A, FAM54B, GLS, IARS2, KIAA0564, LACTB, LETM1, MARC2, MAVS, MFF, MRPL23, MRPL24, MRPL28, MRPL41, MRPL47, MRPS11, MRPS24, MRPS26, MRPS35, MTFR1, POLDIP2, PPOX, PYCR1, PYCR2, SLC25A12, TAMM41, TIMM13, TOMM70A, USP30, VARS2, YME1L1) participate in various aspects of mitochondrial function, but without clear-cut relevance for parthanatos. However, Botham and colleagues also identified one final interaction partner of HtrA2/Omi that is indeed a core component of the parthanatic signaling chain: AIFM1. While initial studies reported that AIFM1 needs to be proteolytically cleaved to fulfil its function in parthanatos [49–52], this was later refuted [53, 54]. Also, to the best of our knowledge, there are no reports of proteolytic cleavage of AIFM1 by HtrA2/Omi. Therefore, it is tempting to speculate that HtrA2/Omi might exert its functions in parthanatos via a protease-independent interaction with AIFM1. Of course, HtrA2/Omi might alternatively control parthanatos by interacting with other, yet-to-be-identified proteins. In any case, it will be of high interest to further elucidate the interrelationships between HtrA2/Omi and AIFM1 in the future. To gain further insight into the mechanisms by which HtrA2/Omi controls parthanatos, it will also be definitely worthwhile to conduct BioID or IP-MS screens in parthanatic cells to identify novel parthanatic mitochondrial interaction partners of HtrA2/Omi.

## Materials and methods

### Reagents

MNNG was purchased from Biosynth Carbosynth (Compton, United Kingdom), olaparib was obtained from Axon Medchem (Groningen, The Netherlands), and birinapant from ChemieTek (Indianapolis, IN, USA). Ucf-101 was provided by Biozol (Eching, Germany), highly purified human recombinant TNF by BASF Bioresearch (Ludwigshafen, Germany), benzyloxycarbonyl-Val-Ala-Asp(OMe)-fluoromethylketone (zVAD-fmk) by Bachem (Bubendorf, Switzerland), and cycloheximide (CHX) by Sigma–Aldrich (Munich, Germany).

siRNAs specific for murine LONP1 (J-054897-09-0002), PMPCA (J-055541-05-0002), DBC-1 (J-047837-05-0002), stathmin (J-041608-05-0002) and the non-targeting siRNA (D-001206-13-20) were obtained from Horizon Discovery (Cambridge, UK).

### Cell lines and cell culture

L929Ts is a TRAIL-sensitive subline derived by prolonged passaging in the laboratory from L929 cells that were originally obtained from ATCC [77]. Immortalized MEF deficient for HtrA2/Omi and their WT counterparts have been described [14], as have PARL-deficient MEF and PARL-deficient MEF reconstituted with PARL [64] (all kindly provided by Thomas Langer and Shotaro Saita, Cologne, Germany). Parental as well as Omi- or PARL-deficient and -reconstituted cells were cultured in Click’s/RPMI 1640 [50/50% (v/v), L929Ts] or DMEM (MEF), supplemented with 10% (v/v) heat-inactivated FBS, 2 mM l-glutamine (only for L929Ts), 50 µg/ml penicillin/streptomycin, and 50 µM β-mercaptoethanol in 0.9% (w/v) NaCl in a humidified incubator with 5% (v/v) CO_2_ at 37 °C.

### Deletion of HtrA2/Omi by CRISPR/Cas9

The CRISPR/Cas9 vector pCMV-U6gRNA-Cas9-2A-GFP containing a gRNA targeting murine HtrA2/Omi (Target ID: MM0000364614, Sigma–Aldrich) was transfected into parental L929Ts cells by Amaxa nucleofection (Lonza, Cologne, Germany) using solution V and program T-20. After transfection, cells were sorted for GFP expression by FACS. Individual clones were isolated and analyzed for loss of HtrA2/Omi expression by Western blot.

### Immunoblots

After harvesting the cells, they were lysed at 4 °C in TNE buffer [50 mM Tris pH 8.0, 150 mM NaCl, 1% (v/v) NP-40, 3 mM EDTA, 1 mM sodium orthovanadate, 5 mM sodium fluoride] containing 10 µg/mL cOmplete protease inhibitor cocktail. Identical amounts of cell protein per lane were resolved by electrophoresis on SDS–polyacrylamide gels. After electrophoretic transfer onto nitrocellulose, reactive proteins were detected with antisera specific for HtrA2/Omi (15775-1-AP, Proteintech, Rosemont, IL, USA), ab75982 (Abcam, Cambridge, UK), or AF1458 (R&D Systems, Minneapolis, MN, USA), LONP1 (ab103809, Abcam), PMPCA (ab140171, Abcam), PARP-1 (9542, Cell Signaling, Danvers, MA, USA), cIAP1 (ALX-803-335-C100, Enzo Life Sciences, Lörrach, Germany), PDXDC1 (21021-1-AP, Proteintech), VPS4B (ab102687, Abcam), DBC-1 (5693, Cell Signaling), moesin (ab151542, Abcam), stathmin (ab52630, Abcam), COXIV (4844, Cell Signaling) and the LumiGLO chemiluminescent substrate (Cell Signaling). Equal loading as well as transfer efficiency was routinely verified for all Western blots by Ponceau S staining and by re-staining the membranes for actin (A1978, Sigma–Aldrich).

### Confocal laser scanning microscopy

Cells were seeded on glass coverslips and grown over night at 37 °C. To visualize mitochondria, cells were incubated with 300 nM MitoTracker™ Orange CMTMRos (ThermoFisher Scientific, Waltham, MA, USA) for 15 min at 37 °C, then washed twice with cold TBS (50 mM Tris pH 7.6, 150 mM NaCl) and fixed in 2.5% (w/v) paraformaldehyde for 10 min on ice. The coverslips were washed two more times in cold TBS and incubated with cold methanol (− 20 °C) on ice for 10 min, followed by two more washes with cold TBS and blocking of nonspecific sites with 1% (w/v) BSA in TBS for 15 min at room temperature and three washes in TBS for 5 min each. Cells were incubated overnight with primary antibody (anti-HtrA2/Omi rabbit polyclonal IgG (15775-1-AP, Proteintech) for parental or HtrA2/Omi-deficient cells, anti-HtrA2/Omi rabbit polyclonal IgG (AF1458, R&D Systems) for HtrA2/Omi-reconstituted cells, or anti-FLAG M2 mouse monoclonal IgG (F1804, Sigma–Aldrich) for PARL-FLAG-reconstituted cells, all 1:200 in TBS with 1% (v/v) BSA), washed four times in TBS (8 min each), incubated for 2 h at room temperature with secondary antibody [goat anti-rabbit IgG (H + L) cross-adsorbed secondary antibody, Alexa Fluor 488 (A-11008, ThermoFisher Scientific) or goat anti-mouse IgG (H + L) cross-adsorbed secondary antibody, Alexa Fluor 488 (A-11001, ThermoFisher Scientific), both 1:1000 in TBS with 1% (v/v) BSA] with addition of Hoechst 33342 dye (1:1000, ThermoFisher Scientific) to stain the nuclei, washed four times in TBS (8 min each), once in Milli-Q water (2 min) and then analyzed using a Zeiss LSM 510 confocal laser scanning microscope (Zeiss, Oberkochen, Germany). If required, contrast and brightness of final digital images were adjusted with LSM Image Browser 4.2.0.121 software (Zeiss), always applying identical settings to all subpanels of one image.

### Reconstitution of HtrA2/Omi-deficient cells

Stably reconstituted HtrA2/Omi-deficient MEF expressing WT HtrA2/Omi or protease-inactive HtrA2/Omi S306A were obtained by nucleofection of pcDNA3.1 encoding WT HtrA2/Omi or HtrA2/Omi S306A (constructs kindly provided by Edward Mocarski, Atlanta, GA, USA), cotransfection with pcDNA3.1 Zeo and subsequent selection with 250 µg/ml Zeocin (ThermoFisher Scientific). HtrA2/Omi-deficient L929 cells were reconstituted by nucleofection with pcDNA3.1 encoding WT HtrA2/Omi and selected with 1000 µg/ml G418 (Sigma–Aldrich). Single clones were isolated and checked for expression of the respective construct by Western blot.

### Flow cytometric analysis of membrane integrity

Following treatment, both detached and adherent cells were collected by centrifugation. The cells were resuspended in PBS/5 mM EDTA containing 2 μg/ml propidium iodide (PI), and the red fluorescence was measured on a FACSCalibur (BD Biosciences, Heidelberg, Germany) or a MACSQuant X (Miltenyi Biotec, Bergisch Gladbach, Germany) flow cytometer. Representative flow cytometry pseudocolor dot plots for all performed analyses of membrane integrity are provided in Supplementary Fig. 3.

### Statistical analysis

Data were tested for normality using the Shapiro–Wilk test where appropriate. Normally distributed groups were compared by two-tailed unpaired Student’s *t* test, otherwise by Mann–Whitney test using GraphPad Prism 9 (GraphPad Software, La Jolla, CA, USA). *p* values < 0.05 were considered statistically significant (*p < 0.05; **p < 0.01; ***p < 0.001).

### Imaging of cell morphology

For documentation of cell morphology, images from unfixed cells were obtained using a NYONE automated cell imager (Synentec, Elmshorn, Germany).

### RNA interference

WT L929Ts cells and MEF were transfected with 150 pmol siRNA by Amaxa nucleofection (Lonza), using the Cell Line Nucleofector Kit V and program T-020 (L929Ts cells) or the Basic Nucleofector Kit for Primary Mammalian Fibroblasts and program A-023 (MEF), and incubated for 48 h at 37 °C before further analysis.

### Preparation of mitochondrial and cytosolic fractions

Highly enriched mitochondrial and cytosolic fractions were generated with the Cell Fractionation Kit - Standard (ab109719, Abcam) according to the instructions of the manufacturer.

### Mass-spectrometric screen for potential downstream mediators of HtrA2/Omi by N‑terminomics (HYTANE)

Highly enriched mitochondrial fractions from untreated and MNNG-treated WT and HtrA2/Omi KO MEF were prepared in biological triplicates with the Mitochondria Isolation Kit for Cultured Cells with Dounce homogenizer (ab110171, Abcam) following the instructions of the manufacturer. To 200 µg of each fraction, SDS was added [1% (w/v) final]. The samples were reduced with Tris(2-carboxyethyl)phosphine hydrochloride (5 mM final) for 30 min at 60 °C and alkylated with iodoacetamide (10 mM final) at room temperature for 30 min. The reaction was quenched with dithiothreitol (20 mM final). Samples were precipitated with chloroform/methanol and brought up in 6 M guanidine HCl in HEPES (200 mM, pH 7) to resuspend the pellet and then diluted to 3 M guanidine HCl. An aliquot of each sample was taken, diluted 1:10 with water and a bicinchoninic acid assay protein measurement was performed. 35 µg of each sample was labelled with an individual tandem mass tag channel which was dissolved in DMSO and mixed 1:1 with sample. A total of 0.6 mg of protein across all channels was labelled. The samples were left to react for 1 h at 25 °C. The labelling reaction was quenched with 8 µl of 5% (w/v) hydroxylamine for 30 min. All channels were combined and the sample was precipitated with ethanol. The pellet was washed with ice-cold ethanol, re-dissolved in 4 M guanidine HCl and then diluted to a final concentration of 0.8 M. The sample was digested with 10 µg of trypsin (20:1 ratio of protein to enzyme) and left to digest overnight at 37 °C. The sample was then cleaned using a C-18 column and eluted with 80% (v/v) acetonitrile (ACN) in 0.1% (v/v) trifluoroacetic acid (TFA). The new N-termini generated as a result of the trypsin digestion were depleted using HYTANE. The sample was redissolved in HEPES buffer (pH 7) before hexadecanal (500 µl, 10 mg/ml) in isopropanol was added along with 20 mM sodium cyanoborohydride and the reaction left for 2 h at 50 °C followed by incubation at 37 °C overnight. A fresh aliquot of sodium cyanoborohydride was added (20 mM), the sample was dried down, resuspended in loading buffer (3% (v/v) ACN in 0.1% (v/v) TFA) and cleaned with a C-18 column. The sample was dried down and stored at − 20 °C prior to analysis.

For liquid chromatography/mass spectrometry (MS) measurements, samples were injected in duplicate on a Dionex Ultimate 3000 nano-UHPLC coupled to a Q Exactive mass spectrometer (Thermo Scientific, Bremen, Germany). The samples were washed on a trap column (Acclaim PepMap 100 C-18, 5 mm × 300 μm, 5 μm, 100 Å, Dionex) for 4 min with 3% (v/v) ACN/0.1% (v/v) TFA at a flow rate of 30 μl/min prior to peptide separation using an Acclaim PepMap 100 C-18 analytical column (50 cm × 75 μm, 2 μm, 100 Å, Dionex). A flow rate of 300 nl/min using eluent A [0.05% (v/v) formic acid (FA)] and eluent B [80% (v/v) ACN/0.04% (v/v) FA] was used for gradient separation (180-min gradient, 540% B). Spray voltage applied on a metal-coated PicoTip emitter (10 μm tip size, New Objective, Woburn, MA, USA) was 1.6 kV, with a source temperature of 250 °C. Full scan MS spectra were acquired between 300 and 2000 m/z at a resolution of 70,000 at m/z 400. The ten most intense precursors with charge states greater than 2 + were selected with an isolation window of 1.4 m/z and fragmented by higher energy collisional dissociation with normalized collision energies of 35 at a resolution of 17,500. Lock mass (445.120025) and dynamic exclusion (30 s) were enabled.

For database search and statistics, The MS raw files were processed by Proteome Discoverer 2.2.0.388 (ThermoFisher Scientific) and MS/MS spectra were searched using the Sequest HT algorithm against a database containing common contaminants and a mouse database. The enzyme specificity was set to semi-ArgC with two missed cleavages allowed. An MS1 tolerance of 10 ppm and a MS2 tolerance of 0.02 Da was implemented. Oxidation (15.995 Da) of methionine residues, acetylation (42.011 Da) and TMT6plex (229.163 Da) on the peptide N-terminus was set as a variable modification while carbamidomethylation (57.02146 Da) on cysteine residues and TMT6plex on lysine residues was set as a static modification. Technical injection replicates were set as fractions. Minimal peptide length was set to 6 amino acids and the peptide false discovery rate (FDR) was set to 1%. Normalized, scaled abundance ratios (to 1) from Proteome Discoverer were exported, log_2_-transformed and statistical analysis (ANOVA) performed in Perseus (Perseus_1.6.10.43). To compensate for the multiple testing hypothesis, a permutation-based false discovery rate (FDR) of 0.05 (5%) and an s0 value of 0.1 (for analysis of protein abundances) or an s0 value of 0 (for analysis of protease-generated neo-N termini) were utilized. A post-hoc Tukey honestly significant difference (HSD) test 0.05 (5%) was implemented to determine the significant pairs in the ANOVA. Additionally, for analysis of protein abundances, in a less stringent, alternative approach, ANOVA was performed on scaled abundances without log_2_ transformation, and groups were additionally compared pairwise by Student’s *t* test, using a p-value of less than 0.05 and a fold change of ± two standard deviations from the median as a cut-off.

## Supplementary Information

Below is the link to the electronic supplementary material.

Supplementary file1 (TIF 18078 KB)

Supplementary file2 (TIF 20145 KB)

Supplementary file3 (PDF 76894 KB)

Supplementary file4 (DOCX 85 KB)

Supplementary file5 (XLSX 116 KB)

Supplementary file6 (XLSX 83 KB)

Supplementary file7 (XLSX 228 KB)

Supplementary file8 (XLSX 156 KB)

## Data Availability

All data generated or analyzed during this study are included in this published article and its supplementary information files.

## Abbreviations

AIFM1: Apoptosis inducing factor mitochondria associated 1
BioID: Proximity-dependent biotin identification
CHX: Cycloheximide
DBC-1: Deleted in breast cancer 1
HtrA: High Temperature Requirement A
HYTANE: HYdrophobic Tagging-Assisted N-termini Enrichment
IAPs: Inhibitor of apoptosis proteins
IMS: Mitochondrial intermembrane space
IP-MS: Immunoprecipitation coupled with mass spectrometry
KO: Knock-out
LONP1: Lon peptidase 1, mitochondrial
MEF: Mouse embryonic fibroblasts
MIF: Macrophage migration inhibitory factor
MNNG: 1-Methyl-3-nitro-1-nitrosoguanidine
MPP: Mitochondrial processing peptidase
PAR: Poly(ADPribose)
PARL: Presenilins-associated rhomboid-like protein, mitochondrial
PARP-1: PAR polymerase 1
PDXDC1: Decarboxylase domain-containing protein 1
PI: Propidium iodide
PMPCA: Peptidase, mitochondrial processing subunit alpha
TNF: Tumor necrosis factor
TRAIL: TNF-related apoptosis-inducing ligand
VPS4B: Vacuolar protein sorting 4 homolog B
WT: Wild-type
zVAD-fmk: Benzyloxycarbonyl-Val-Ala-Asp(OMe)-fluoromethylketone
