# Supplementary material for: Protease-independent control of parthanatos by HtrA2/Omi
Source: Cell Mol Life Sci. 2023 Aug 18;80(9):258. doi: 10.1007/s00018-023-04904-7 (PMC10439076; doi:10.1007/s00018-023-04904-7)
Supplement: Supplementary file 3 — Supplementary file3 (PDF 76894 KB) [file 18_2023_4904_MOESM3_ESM.pdf]

Supplementary Figure 3:
Representative flow cytometry pseudocolor dot plots for all analyses of membrane integrity shown in the main manuscript.

Figure 1

C

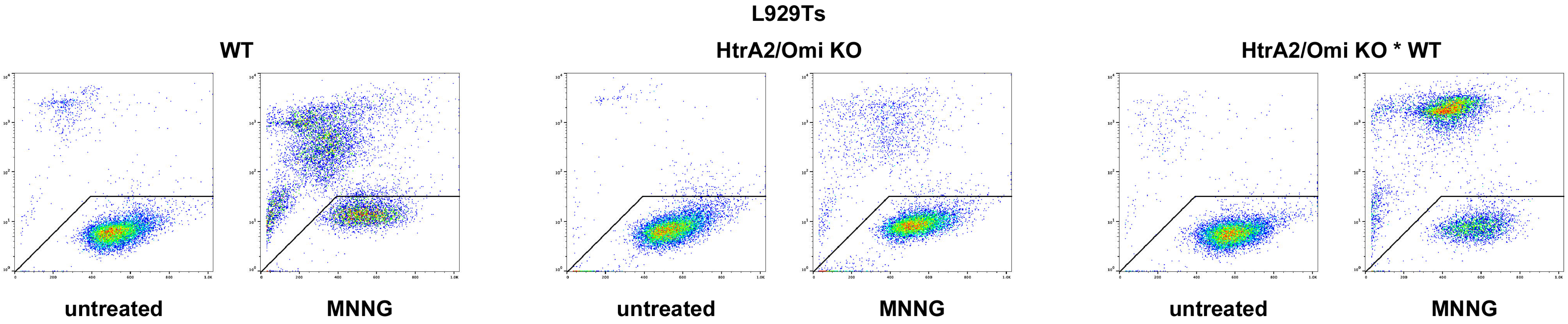

D

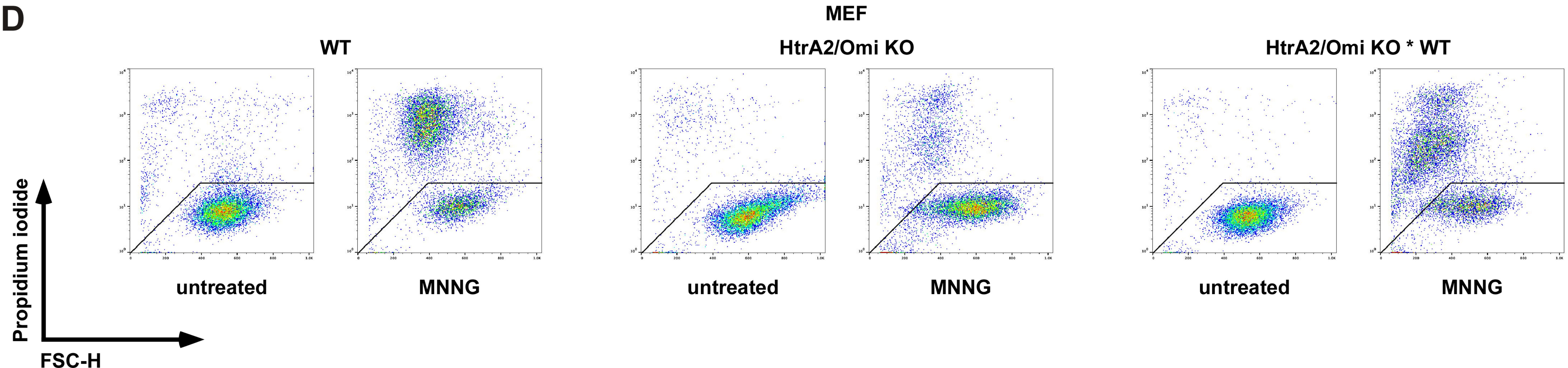

Figure 2

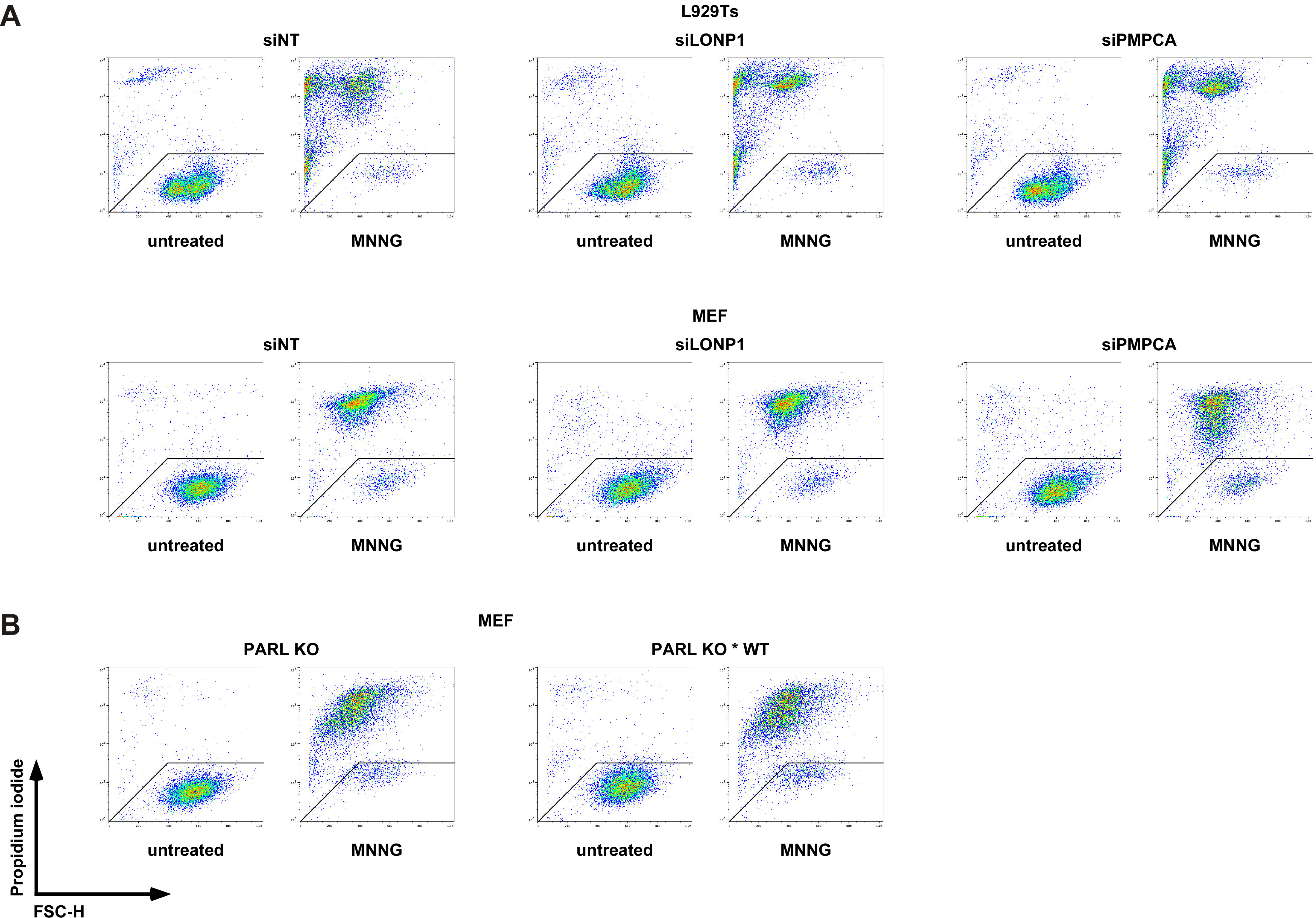
A
L929Ts
siNT
siLONP1
siPMPCA
untreated
MNNG
untreated
MNNG
untreated
MNNG
MEF
siNT
siLONP1
siPMPCA
untreated
MNNG
untreated
MNNG
untreated
MNNG
B
MEF
PARL KO
PARL KO * WT
untreated
MNNG
untreated
MNNG
Propidium iodide
FSC-H

Figure 3

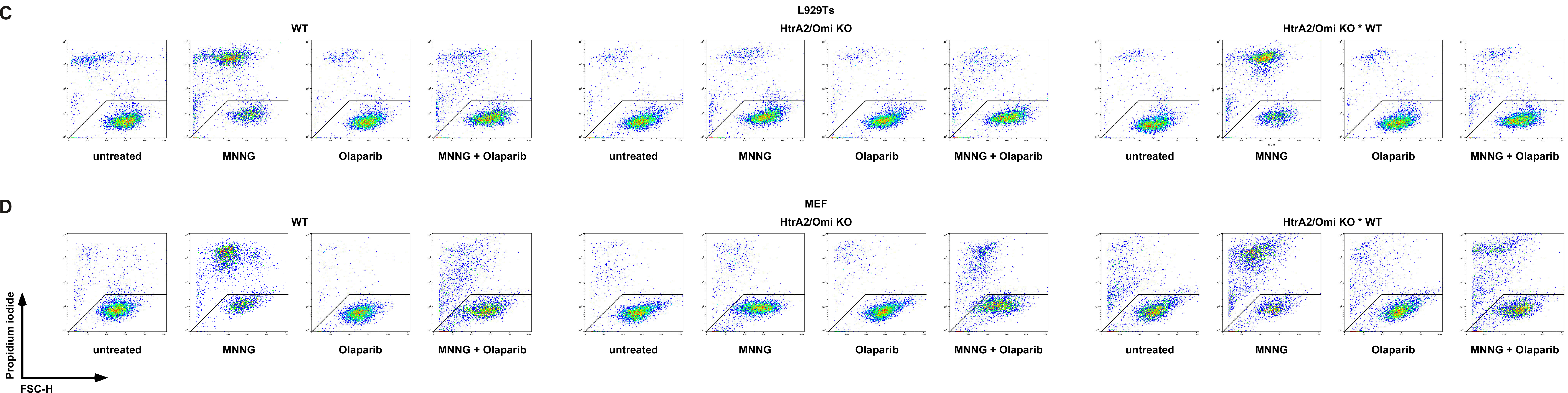
C
L929Ts
WT
HtrA2/Omi KO
HtrA2/Omi KO * WT
untreated
MNNG
Olaparib
MNNG + Olaparib
untreated
MNNG
Olaparib
MNNG + Olaparib
untreated
MNNG
Olaparib
MNNG + Olaparib
D
MEF
WT
HtrA2/Omi KO
HtrA2/Omi KO * WT
untreated
MNNG
Olaparib
MNNG + Olaparib
untreated
MNNG
Olaparib
MNNG + Olaparib
untreated
MNNG
Olaparib
MNNG + Olaparib
Propidium iodide
FSC-H

Figure 4

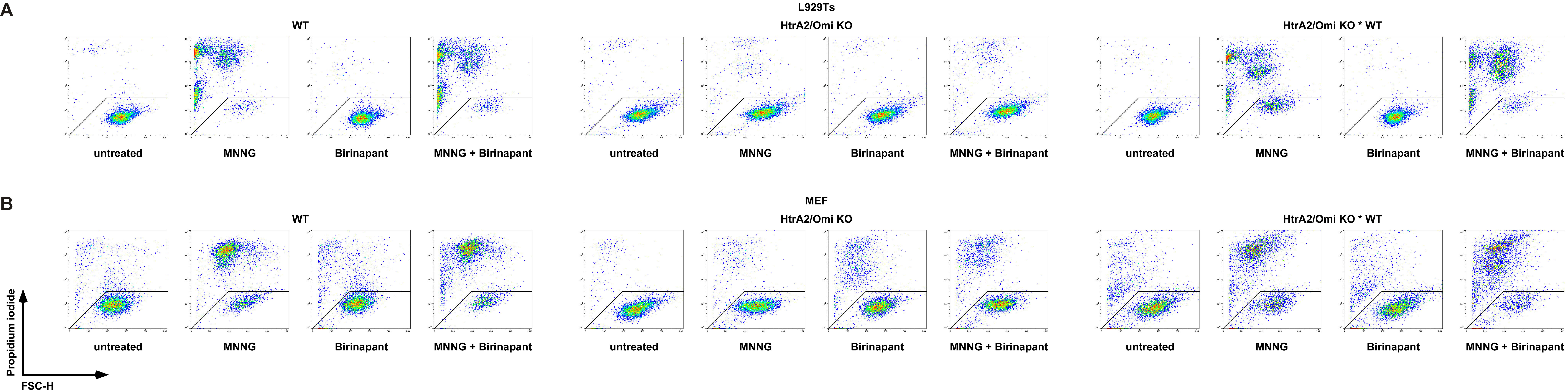
A
L929Ts
WT
HtrA2/Omi KO
HtrA2/Omi KO * WT
untreated
MNNG
Birinapant
MNNG + Birinapant
untreated
MNNG
Birinapant
MNNG + Birinapant
untreated
MNNG
Birinapant
MNNG + Birinapant
B
MEF
WT
HtrA2/Omi KO
HtrA2/Omi KO * WT
untreated
MNNG
Birinapant
MNNG + Birinapant
untreated
MNNG
Birinapant
MNNG + Birinapant
untreated
MNNG
Birinapant
MNNG + Birinapant
Propidium iodide
FSC-H

Figure 5

B

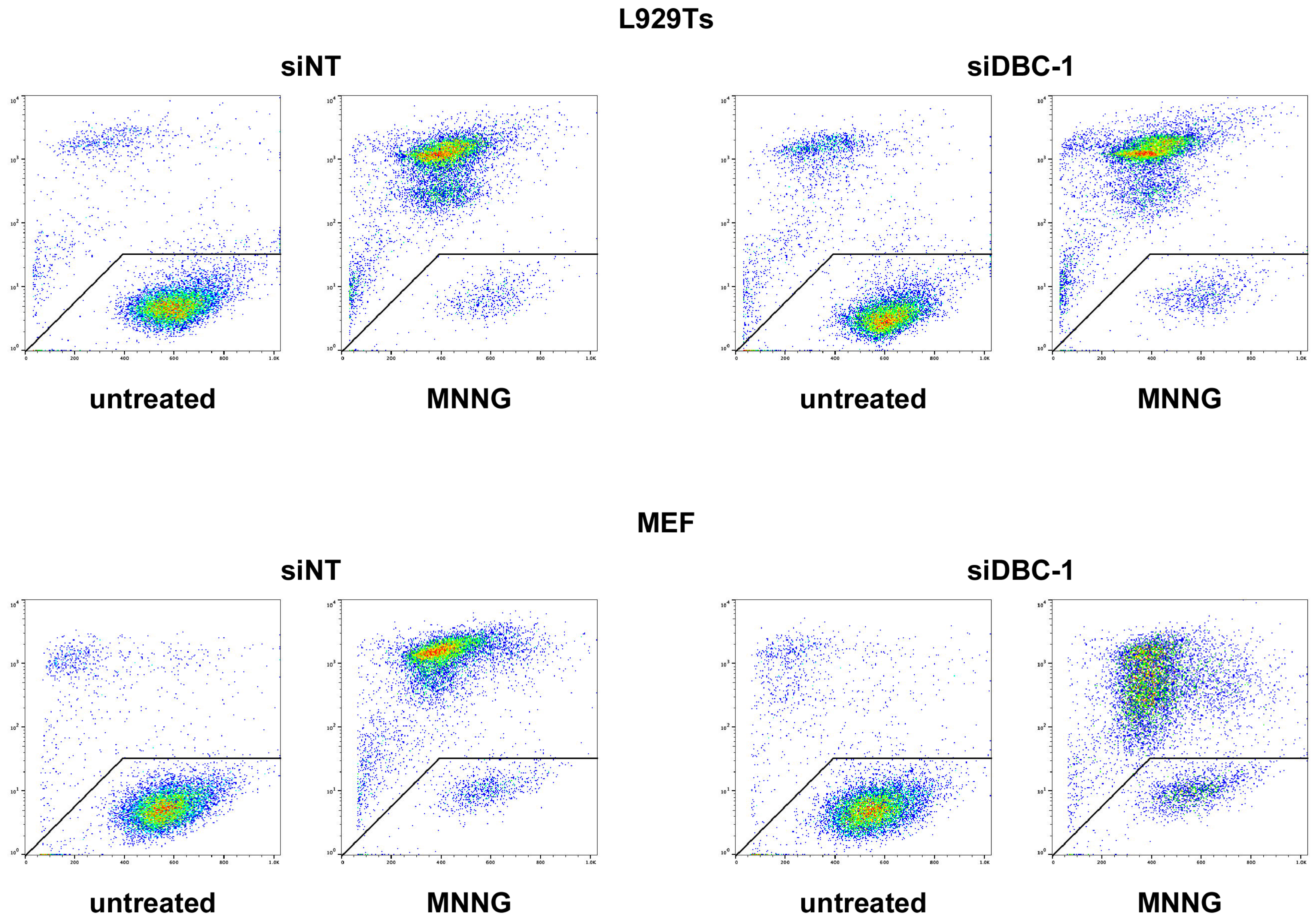
L929Ts
siNT
siDBC-1
untreated
MNNG
untreated
MNNG
MEF
siNT
siDBC-1
untreated
MNNG
untreated
MNNG

D

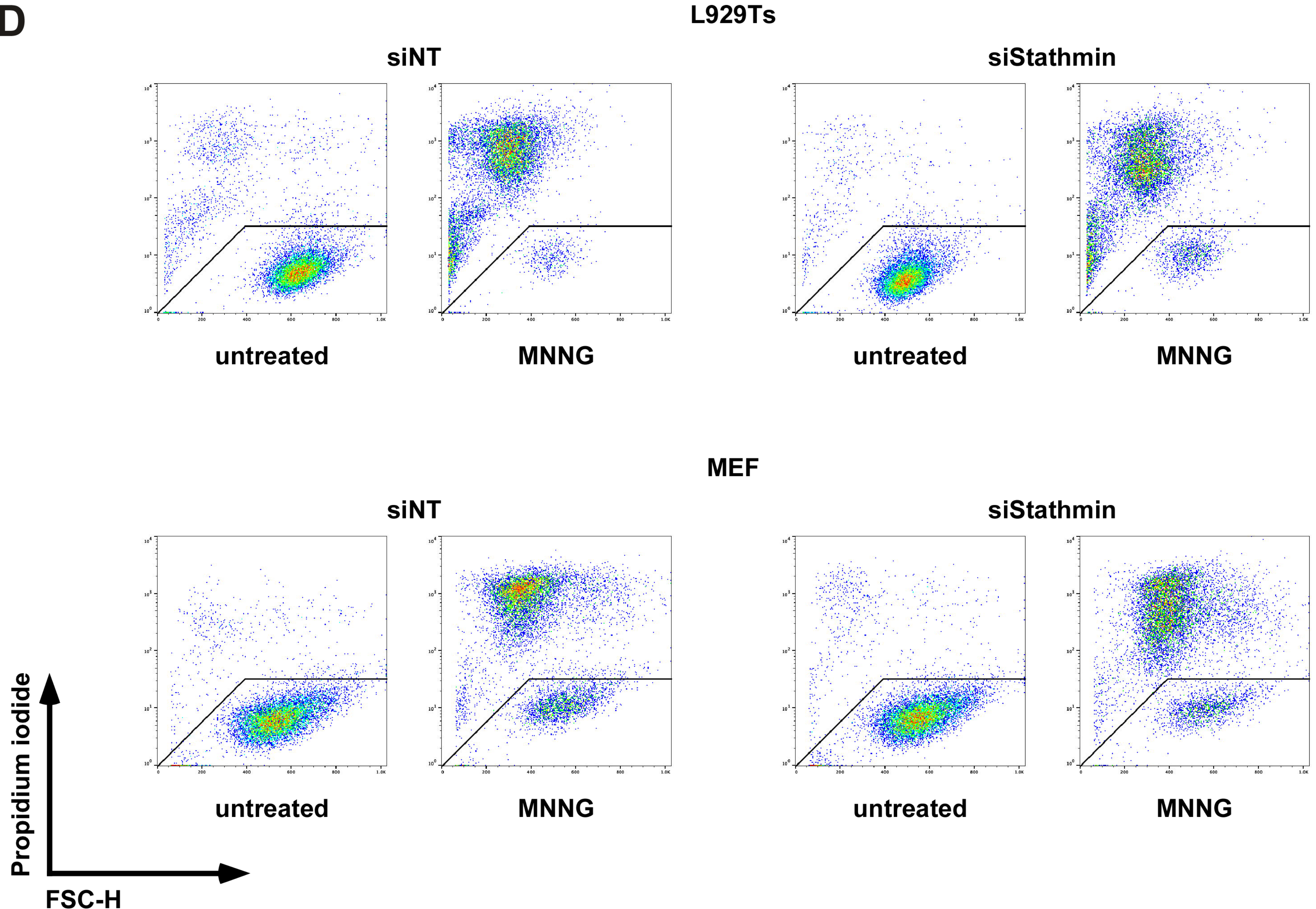
L929Ts
siNT
siStathmin
untreated
MNNG
untreated
MNNG
MEF
siNT
siStathmin
untreated
MNNG
untreated
MNNG
Propidium iodide
FSC-H

Figure 7

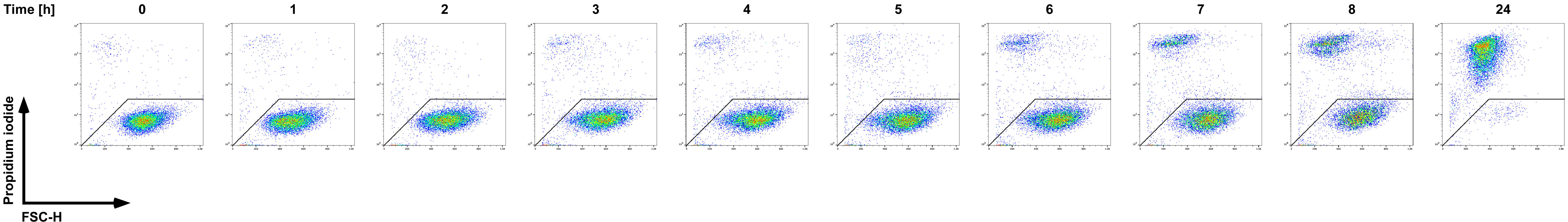

Figure 9

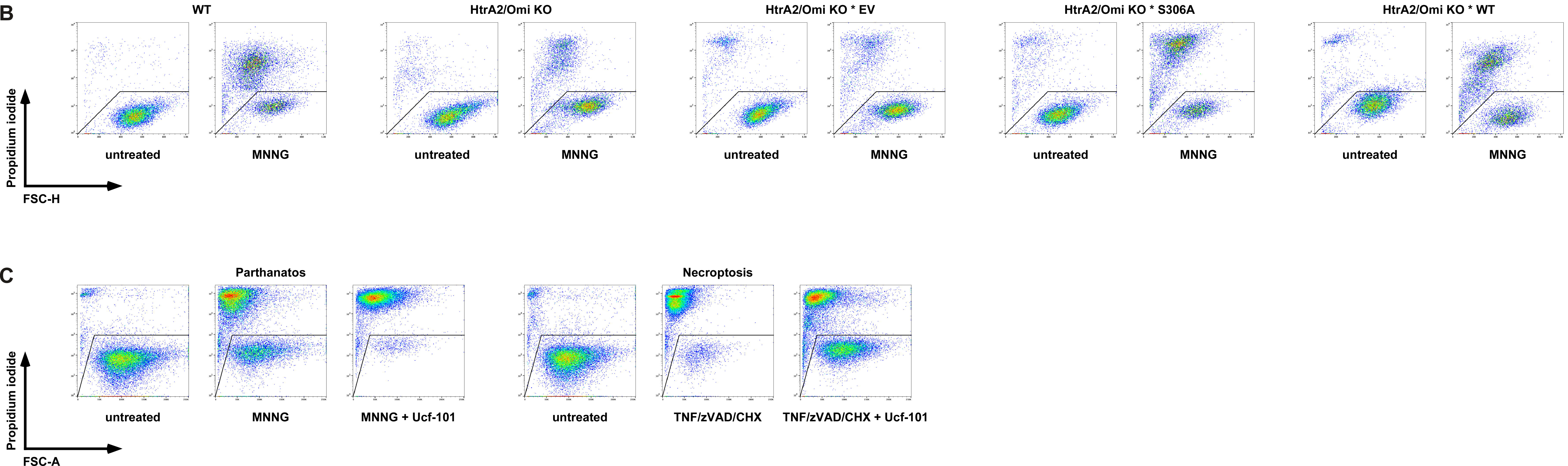
B
WT
HtrA2/Omi KO
HtrA2/Omi KO * EV
HtrA2/Omi KO * S306A
HtrA2/Omi KO * WT
untreated
MNNG
Propidium iodide
FSC-H
C
Parthanatos
Necroptosis
untreated
MNNG
MNNG + Ucf-101
untreated
TNF/zVAD/CHX
TNF/zVAD/CHX + Ucf-101
Propidium iodide
FSC-A
