# Supplementary material for: Protease-independent control of parthanatos by HtrA2/Omi
Source: Cell Mol Life Sci. 2023 Aug 18;80(9):258. doi: 10.1007/s00018-023-04904-7 (PMC10439076; doi:10.1007/s00018-023-04904-7)
Supplement: Supplementary file 4 — Supplementary file4 (DOCX 85 KB) [file 18_2023_4904_MOESM4_ESM.docx]

# Supplementary Figure Legends

Supplementary Figure 1: HtrA2/Omi is not released from mitochondria into the cytosol during parthanatos. (A) HtrA2/Omi KO * WT L929Ts cells and (C) WT and (D) HtrA2/Omi KO * WT MEF were left untreated or stimulated with 0.5 mM MNNG for 15 min and further incubated for the indicated times with fresh medium without MNNG before they were analyzed by immunofluorescence microscopy. HtrA2/Omi is indicated by green fluorescence (top left), mitochondria (red) were stained with MitoTracker Orange (top right), cell nuclei (blue) were stained with Hoechst 33342 (bottom left), and an overlay of all stainings is shown at the bottom right. Scale bars, 10 µm. (B) WT MEF were left untreated (0) or treated with 0.5 mM MNNG for 15 min and further incubated with fresh medium without MNNG for the indicated times. Subsequently, mitochondrial and cytosolic fractions were prepared and the presence of HtrA2/Omi in these fractions was examined by Western blot. Detection of the mitochondrial marker COXIV was used to ensure that the cytoplasmic fractions were free from mitochondrial contaminations. Given its tight association with mitochondria [78], detection of actin served as a loading control for both mitochondrial as well as cytosolic fractions.

Supplementary Figure 2: HtrA2/Omi is released from mitochondria upon induction of apoptosis. (A) WT and (B) HtrA2/Omi KO * WT L929Ts cells and MEF (C, D) were left untreated or pretreated with 10 (L929) or 1 µg/ml (MEF) CHX for 30 min and, after addition of 100 ng/ml TNF, further incubated to induce apoptosis for the indicated times before they were analyzed by immunofluorescence microscopy. HtrA2/Omi is indicated by green fluorescence (top left), mitochondria (red) were stained with MitoTracker Orange (top right), cell nuclei (blue) were stained with Hoechst 33342 (bottom left), and an overlay of all stainings is shown at the bottom right. Scale bars, 10 µm.

Supplementary Figure 3: Representative flow cytometry pseudocolor dot plots for all analyses of membrane integrity shown in the main manuscript. The gate separates cells counted as PI-positive with loss of membrane integrity (upper area) from cells counted as still having an intact membrane. The flow cytometry data for Figure 8C were generated on a MACSQuant X flow cytometer and are shown as PI vs. FSC-A, all other data on a FACSCalibur device (shown as PI vs. FSC-H).
